# Comparison of CPG’s for the diagnosis, prognosis and management of non-specific neck pain: a systematic review

**DOI:** 10.1186/s12891-019-2441-3

**Published:** 2019-02-14

**Authors:** Pulak Parikh, Pasqualina Santaguida, Joy Macdermid, Anita Gross, Arshia Eshtiaghi

**Affiliations:** 1 School of Rehabilitation Science, McMaster University, Hamilton, ON L8S 1C7 Canada; 2 Department of Health Research Methods, Evidence and Impact (HEI), Faculty of Health Sciences, McMaster University, Hamilton, ON Canada; 3 Hand and Upper Limb Centre, St. Joseph Hospital, London, ON Canada; 4 Clinical Epidemiology & Biostatistics, McMaster University, Hamilton, ON Canada; 5 Department of Health Science, McMaster University, Hamilton, ON Canada

**Keywords:** Clinical practice guidelines, Neck pain populations, Systematic review

## Abstract

**Background:**

Neck pain (NP) is a very common musculoskeletal condition with potential for a high burden in disability and length of disorder. Clinical practice guidelines (CPG) give recommendations to clinicians for providing optimal care for patients however best practice recommendations are often contradictory. The purpose for this review was to conduct a SR of CPGs to assess the management recommendations for NP (diagnosis, treatment, prognosis, imaging).

**Methods:**

Standard SR methodology was employed including a grey literature search (including the National Guideline Clearing House). Medline, Cinahl, Embase, ILC, Cochrane, Central, and Lilacs were searched from 1995-to March 2018. Two raters evaluated all citations and a third rater resolved any disagreements. The AGREE II was used to assess risk of bias of each CPG. Data was extracted and included CPG purpose, type of NP problem and clinical recommendations. The AGREE II critical appraisal tool was used to assess risk of bias of each CPG.

**Results:**

From 640 articles, 241 were available for screening. A total of 46 guidelines were selected. CPG’s were categorized by the NP population (General NP, whiplash, interventional, headache and risk for vertebral insufficiency) and type of clinical aim (diagnosis, prognosis, treatment, imaging). Each clinical NP population had a large overlap of clinical aims presented. The CPGs were directed to a variety of clinicians that included physicians, physiotherapists and chiropractors. Results suggest heterogeneity in CPG recommendations within each clinical aim. CPG characteristics accounting for these differences are outlined.

**Conclusion:**

The majority of CPGs were developed for general NP that focused on treatment recommendations, with fewer number aimed at recommendations for diagnosis, prognosis, and outcomes. Heterogeneity of recommendations within the categories were noted as were potential factors associated with these differences, including CPG quality as assessed by the AGREE II.

**Electronic supplementary material:**

The online version of this article (10.1186/s12891-019-2441-3) contains supplementary material, which is available to authorized users.

## Background

Neck pain (NP) is the most common musculoskeletal pathology second only to low back pain [1]. It is the fourth largest contributor to global disability with its prevalence ranging between 30 to 71% of the general population [2, 3]. Two thirds of adults are affected by NP at some time in their lives [4]. Not only does it have a potential for a high burden in disability most people with NP do not experience a complete resolution of symptoms [3, 5]. The economic burdens include cost of healthcare, reduced work productivity, work absenteeism and insurance that is estimated at 33.6 million annually [2, 5] .

In efforts to decrease the amount of practice variation for NP, many guidelines have been developed to improve efficiency and effectiveness. Clinical practice guidelines (CPGs) are defined by the Institute of Medicine as “recommendations that intend to optimize patient care that are informed by systematic review (SR) of the evidence with an assessment of harms and benefits of alternative care options” [6]. They are intended to give best practice guidance and recommendations to clinicians for providing optimal care for patients. However, there has been mounting criticism that recommendations across different CPGs are often contradictory. Due to a large number of guidelines available for NP, clinicians often find it challenging to determine which guidelines are credible and of high quality. Many of the commonly used guidelines for NP have been questioned for their methodological quality, validity and reliability [7–9]. Furthermore, evidence for the treatment of mechanical NP is often of low quality and conflicted [10, 11], possibly resulting in a difference between the recommendations. Despite this, patients that receive adherent care in relation to CPG’s for NP have had significantly lower visits to health care providers, decreased use of prescription medication and fewer diagnostic images [12, 13]. As such, the pursuit of guideline development, if originating from a strong scientific and pragmatic foundation is highly promising to the successful identification and treatment of neck pain.

To date, no previous research has systematically summarized and appraised all relevant CPG’s for the management of NP. The purpose of this SR was not only to summarize the existing evidence regarding all CPGs regarding NP but also to provide a “panoramic view” of the CPG literature with an appraisal of the methodological quality. In the context of NP, an SR is ideal to provide the evaluation of the evidence from many different clinical settings, professions that treat NP and different countries where guidelines are developed. NP’s widespread frequency and reoccurrence can cause differentiating levels of pain and disability, highlighting the need for a global summary of findings.

## Methods

### Search strategy

This SR was an update of an existing review that was updated for CPG’s only [14]. A search strategy was developed with consultation from a health sciences librarian that included reviews from 2000 to 2012. An update was systematically undertaken from January 2012 to March 2018 for CPGs only. Details of the search strategy are outlined in detail here [14]. The following databases were searched: Medline, PubMed, Embase, Cochrane Database, CINAHL, ILC, LILACS and CENTRAL along with the grey literature. The grey literature included but was not limited to the National Guideline Clearinghouse, Canadian Medical Association, National Institute of Health and Clinical Excellence (NICE) Guidance, NICE pathways, World Health Organization and the American College of Physicians Clinical Recommendations. Search terms used within the databases for all areas in the overview of reviews across different clinical areas for the management of NP can be found here (Additional file 1: Appendix A).

### Selection of CPGs

CPG’s focusing on any form of management of neck pain were eligible. CPGs are defined as systematically developed statements to assist practitioners and patient decisions for specific clinical circumstance; they can be developed by local, regional, national or international groups or affiliated governmental organizations [14]. Consensus statements are similar but reflect a different methodology for deriving recommendations. Clinical algorithms are also variable in how they present recommendations and are often included within CPG’s. Therefore, consensus statements and algorithms were only included if they were a part of a CPG. Articles had to include populations that had any type of NP.

Citations identified within the search were uploaded into a SR software (DistillerSR) and screening using a standardized form for eligibility. SR and CPGs were eligible but narrative reviews, commentaries, editorials were excluded. Two independent raters screened the articles at titles and abstract and full text with conflicts resolved between the screeners. If the screeners were unable to resolve conflicts, a senior investigator made the final decision for eligibility.

The following criteria were used to include CPGs: 1) All CPGs that included recommendations 2) English language; 3) Diagnosis of non-malignant NP that included adult (> 18 years of age) populations; 4) NP defined as pain from the occiput to upper thoracic spine (T1 to T6, mid upper back) and can include upper regions of the torso or shoulder area; 5) General musculoskeletal or chronic pain guidelines that could potentially include NP populations; Articles were excluded if they met the following criteria: 1) narrative reviews or articles that only contain consensus statements or algorithms; 2) included children (< 18 years of age); 3) trauma associated with fracture or head injury; 4) definite or possible long tract neurological signs (i.e. myelopathies); 5) NP caused by other pathological entities (i.e. tumor, infections); 6) headache not of cervical origin but associated with the neck (i.e. migraine headache).

### Critical appraisal of guidelines

Appraisal of the eligible guidelines was conducted using the Appraisal of Guidelines for Research and Evaluation version II (AGREE II). It is the most commonly used guideline appraisal tool [15] that includes 23 criteria (items) organized over 6 domains and two overall assessments. The items within the first 23 categories are rated on a 7-point scale (strongly agree to strongly disagree). The overall guideline quality is rated on a 7-point scale (lowest possible quality to highest possible quality). A second overall assessment consists of a recommendation provided on whether to use the guideline in practice or not (recommendation for use: yes, yes with modifications, no). Both overall assessment criteria should consider the 23 items evaluated beforehand and the resulting domain scores but should not be calculated from them.

Two to four independent researchers appraised all included guidelines. Each investigator was trained using the web tutorials provided on the AGREEPLUS website in addition to individual one on one sessions with a senior researcher. Discussions were held between the investigators with regards to overall guideline quality. Final grading was determined using the AGREE II scoring system for each domain as a %. The overall guideline quality scores were based out of 7 with recommendations for use in practice had three options (yes, yes with modifications or no).

### Data extraction

One researcher extracted guideline data and a second checked the data. Extracted elements were organized in tabular format and included demographic information (country of origin, professional composition panel), aim of CPG recommendations (diagnosis, prognosis, treatment, imaging) and specific recommendations grouped according to the CPG intent of diagnosis, prognosis, interventions and imaging.

Recommendations from each of the guidelines were organized via tables to facilitate comparison between them based upon their intention. They were divided into those that 1) recommended specific course of action 2) recommended against a specific course of action 3) did not note any course of action (for example, no recommendation for duration of treatment) 4) explicitly noted that there was no evidence to support of refute the recommendation.

## Results

Our search yielded 3082 citations from our databases and 20 from other grey literature sources for a total of 3102 (Additional file 2: Appendix B). We removed 224 titles and abstracts that were duplicates. A total of 2880 citations were screened at title and abstract. Of those, 2239 (76%) did not meet the eligibility criteria. This left 641 articles for which full text was obtained for further screening. Of those, 244 CPGs were included. Two independent reviewers further screened the 244 CPGs that remained. We found 46 (1.5%) guidelines that were deemed admissible and included for appraisal and review.

The majority of guidelines originated from Australia, Canada, United States and the UK (93%) (Additional file 3: Appendix C). CPG authors and committee members included physicians, physiotherapists, chiropractors, nurses, osteopaths, massage therapists and academics. Medical doctors authored the majority of interventional guidelines (*N* = 8) and in contrast physiotherapists and chiropractors were the primary leads in CPG’s for the whiplash, NP and headache guidelines (*N* = 28).

### Guideline quality

**Fig. 1:**
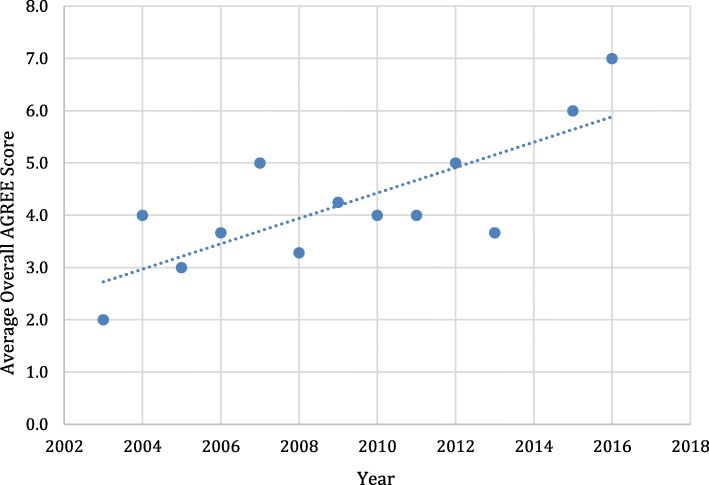
AGREE II Scores Over Time

The overall quality of the included guidelines varied greatly (Additional file 4: Appendix D). When comparing all the guidelines there was a linear progression in the average of the total scores over the years (Fig. 1). Newer guidelines scored higher overall (r^2^ = .53). Guidelines scored poorly in domain-5 (applicability), domain-6 (editorial independence) and domain-3 (rigor of development). With the exception of five, guidelines did not adequately describe facilitators and barriers to their application, give recommendations on how to be put into practice and outline the potential resource implications of applying the recommendations given [16–20]. Guidelines also lacked a description of the funding bodies and their influence on the content of the guideline and competing interests development group [21–42]. Over half of the guidelines had major limitations with systematic search methods used for evidence, criteria of selecting evidence and adequate descriptions of the strengths and limitations of the body of evidence [21, 22, 25, 26, 29, 31, 32, 34–37, 39, 42–50]. The methods for formulating the recommendations in these guidelines were often not described along with a (lack of) link given to the supporting evidence. Domains-1,2 and 4 scored better for all the guidelines throughout the diagnoses.

## General neck pain guidelines

A total of 20 guidelines that had a specific diagnosis of NP and associated disorders were grouped (Table 1). Publication dates spanned from 2003 to 2018. These included three guidelines that referenced cervical radiculopathy [45, 51]. All guidelines were authored by either physician, physiotherapist or chiropractor groups with the exception of one [38]. The majority of the guidelines (17/20) failed to identify barriers and facilitators to implementation, strategies to improve uptake and outline resource implication of applying the guideline [21, 22, 33, 36–45, 51–53].. Half of the guidelines (10/20) showed minimal evidence of editorial independence as the majority of guidelines in this group were funded by groups associated with their authors [21, 22, 33, 36–42]. The AGREE scores improved for guidelines published from 2012 onwards (Fig. 1). Five guidelines in this group were deemed to have a high overall AGREE II score [16, 45, 51, 52, 54].

**Table 1:** AGREE II scores General Neck Pain Guidelines

Author	Year	Scope and Purpose	Stakeholder involvement	Rigor of Development	Clarity of Presentation	Applicability	Editorial Independence	Overall Score
French [21]	2003	53.7	53.7	16.7	44.4	22.2	2.8	2
ANAES [22]	2003	50.0	52.8	19.8	36.1	22.9	8.3	2
Anderson-Peacock [33]	2005	79.6	63.0	58.3	79.6	40.3	47.2	5
Bussieres [43]	2008	72.2	73.6	40.6	61.1	32.3	72.9	4
Guzman [52]	2008	72.2	83.3	67.7	77.8	39.6	91.7	6
PAC [36]	2009	19.4	22.2	15.6	83.3	20.8	12.5	1
AAMPG [53]	2010	64.8	75.9	53.5	81.5	31.9	58.3	5
New York State Workers [37]	2010	58.3	25.0	23.7	63.9	4.2	4.2	2
Bono [44]	2010	77.8	52.8	40.6	58.3	29.2	79.2	4
Brosseau [38]	2012	61.1	59.3	55.6	48.1	2.8	19.4	4
Monticone [39]	2013	50.0	38.9	44.8	58.3	16.7	8.3	3
SIGN [16]	2013	66.7	77.8	74.0	77.8	68.8	54.2	6
Newman [40]	2013	61.1	50.0	58.3	75.0	35.4	16.7	5
Bryans [41]	2014	69.4	33.3	50.0	66.7	20.8	37.5	3
Colorado Division [42]	2014	61.1	41.7	19.8	83.3	35.4	8.3	3
Bussieres [17]	2016	88.9	55.6	66.7	72.2	62.5	79.2	5
Cote [54]	2016	83.3	86.1	75.0	80.6	52.1	91.7	7
Blanpied [45]	2017	77.8	52.8	40.6	58.3	29.2	79.2	6
Kjaer [51]	2017	97.2	86.1	72.9	83.3	18.8	87.5	7
Bier [56]	2018	72.2	55.6	41.7	77.8	25.0	66.7	4

### Diagnosis

Many CPG’s defined NP differently (Additional file 5: Appendix E). This included their definition for the classification of the duration of NP (acute, sub-acute and chronic) and the severity given for the diagnosis. The majority of guidelines published prior to 2010 did not differentiate on duration of NP [37, 52, 53, 55]. Three guidelines used the Bone and Joint classification (Grade 0–4) for diagnosis comparison [17, 52, 54]. Many of the guidelines did not differentiate on severity or classify NP at all [16, 38, 41, 51, 53, 55, 56]. When guidelines outlined durations for NP, often they conflicted on their time frames. Guidelines outline acute pain as 0–1 month [38, 39, 41] but also as 0–3 months [17, 53, 54, 57]. Of the guidelines that outlined duration of NP, all the guidelines agreed that chronic pain was that which was greater than 3 months [16, 17, 38, 39, 41, 45, 53, 54, 57].

### Prognosis

All guidelines indicative of prognostic factors for general NP outlined neurological symptoms as a factor associated with poor prognosis [37, 39, 42, 43, 52–54, 57] with the exception of the two most recent published guidelines [45, 56] (Additional file 6: Appendix F). Other common factors included age [39, 42, 43, 52–54, 57], psychological factors [39, 42, 43, 45, 52–54, 56, 57], and pre-existing NP [39, 42, 43, 45, 52, 54, 56, 57]. Aside from these, a great deal of heterogeneity existed between the guidelines. Only three of the eighteen guidelines identified pain intensity and disability as poor prognostic indicators for general NP [39, 43, 45].

Various factors were described for psychological influences upon NP prognosis (Additional file 7: Appendix G). Passive coping [39, 42, 43, 52, 54, 56] (defined by strategies that relinquish control of pain to others or to allow other life areas to be affected by pain [58]) and depressive symptoms [37, 39, 42, 43, 54] were the most commonly reported. Other common factors included post-traumatic stress [37, 43, 45, 54], kinesophobia [39, 43, 52, 54] and anxiety [37, 39, 52, 54, 56].

### Intervention

Of all guidelines reporting interventions for NP [16, 17, 37–39, 41, 42, 51–57, 59], all but one did not include active exercise as a beneficial treatment [38]. All but two guidelines recommended manipulation and mobilization as an intervention for NP [38, 53]. The majority of guidelines recommend a use of a combination of exercise, manual therapy and modalities (multi-modal care) [16, 17, 39, 41, 42, 45, 51, 53, 54, 56, 57]. as well as education (such as no rest greater than 3 days and staying active) however four guidelines stated that education was not beneficial [17, 53, 54, 57]. There was quality of inconsistent evidence for commonly used treatments such as electrotherapy, traction, laser therapy, acupuncture and heat/cold (Additional file 8: Appendix H). Of all the guidelines for general NP, half recommended the use of medication alone or in combination with other treatments [16, 17, 37, 39, 42, 51, 54, 55] (the majority authored by physicians). Common medications reported were Non-Opioid Analgesics such as NSAIDS (both oral and topical), Paracetamol and Opioids. The majority of guidelines were against the use of soft collars [17, 37, 39, 42, 52–55, 57]. Pulsed electro-magnetic therapy was recommended by five guidelines as a beneficial treatment [16, 39, 52, 53]. Many interventional recommendations did not change over time as there was consistency for exercise and mobilization and manipulation but a large heterogeneity among all other treatments.

### Diagnostic imaging

Seven guidelines for general NP recommended using the Canadian Cervical Spine Rule in their use of X-ray [43, 45, 52–54, 57, 60] within the acute phase (Additional file 9: Appendix I). Six CPGs did not recommend the use of a routine x-ray for a diagnosis of acute or subacute NP [40, 43, 45, 52–54, 57] however chronic NP was considered differently and the recommendations for x-ray were identified in five CPGs [37, 40, 53, 55, 60]. Magnetic Resonance Imaging (MRI) was considered the best imaging technique for NP [39, 40, 43, 53, 55, 60]. For radicular symptoms both the use of MRI or Computed Tomography (CT) scan were presented as options [37, 39, 40, 45, 52, 53, 55, 60]. Electromyogram (EMG) studies were not recommended because of low diagnostic. CPG’s provided conflicting recommendations for studies such as myelography, bone scan, diagnostic injections and tomography.

## Whiplash

Seven guidelines that exclusively pertained to whiplash were identified (Table 2) [23–25, 61–64]. Publication dates spanned from 2005 to 2014. Whiplash was classified using the Quebec Task Force for all guidelines [65]. Overall scores in this group did not follow the general trend in that their methodological quality did not improve with more current publication dates. All guidelines were authored by either physiotherapist or chiropractor lead groups. Every guideline in this group did not adequately identify barriers and facilitators to implementation, strategies to improve uptake and resource implications of applying the guideline with the exception of one [18]. The majority of the guidelines (5/7) did not demonstrate editorial independence with a lack of neutrality from the funding bodies [23–26, 63]. Many guidelines (3/7) did not outline the process used to gather and synthesize the evidence, the methods used to formulate the recommendations and to update them [25, 26, 46]. Two guidelines were deemed to have high overall AGREE II score [18, 23].

**Table 2:** AGREE II scores Whiplash Guidelines

Author	Year	Scope and Purpose	Stakeholder involvement	Rigor of Development	Clarity of Presentation	Applicability	Editorial Independence	Overall Score
Leigh [46]	2005	55.6	36.1	37.5	36.1	18.8	58.3	3
Mercer [24]	2007	80.6	75.0	62.5	44.4	25.0	8.3	5
TRACsa [23]	2008	75.0	72.2	61.5	80.6	37.5	8.3	6
Davis [25]	2009	55.6	30.6	20.8	41.7	14.6	8.3	2
Bryans [26]	2010	75.0	55.6	33.3	47.2	35.4	12.5	3
Moore [18]	2010	94.4	77.8	92.7	80.6	85.4	70.8	7
MAA [63]	2014	86.1	69.4	54.2	88.9	22.9	20.8	4

### Diagnosis

The definition for the duration of whiplash differentiated greatly among included guidelines. Half defined acute whiplash as 0 to 12 weeks [23, 25, 46, 63] (Additional file 5: Appendix E). Three guidelines defined acute whiplash from less than one to two weeks [24, 26, 64] with a subacute phase from 1 to 12 weeks. Chronic whiplash was defined as greater than 12 weeks by all but one guideline [61]. Every guideline used the Quebec Task Force for classification of whiplash (0 to 4 grades). Four of the eight guidelines recommended the Visual Analog Scale and Neck Disability Index as outcome measures for diagnosis and treatment [23, 25, 46, 63]. A large amount of heterogeneity existed between the recommendations for clinical diagnostic tests and assessment procedures.

### Prognosis

All included guidelines for whiplash indicated that the presence of some psychological factors provided evidence of poor prognosis (Additional file 6: Appendix F). All but one guideline [61] described high initial pain scores as a factor for poor prognosis. Other common factors related to poor prognosis were older age [26, 27, 61, 64], pre-existing NP [25, 26, 46, 64] and high levels of self-reported disability [23, 25, 46, 63]. The guidelines showed conflicted recommendations for other factors that included collision/trauma type, imaging testing and high amounts of health care usage. Other factors commonly reported were cold sensitivity [23, 63], lack of ROM [23, 61, 63] and gender (female) [26, 61, 64].

Many factors were identified as psychological influences associated with poor prognosis following whiplash diagnosis (Additional file 7: Appendix G). The most common cause for poor prognosis was passive coping [18, 23, 26, 61, 63]. Other common factors included depression [18, 26, 46, 63], catastrophization [18, 23, 26, 63] and anxiety [18, 23, 26, 63].

### Intervention

All guidelines agreed that active exercise was the most beneficial intervention for whiplash associated disorders regardless of the duration of symptoms [23, 25, 26, 46, 61, 63, 64]. Manipulation, mobilisation and education were also recommended throughout all grouped guidelines (Additional file 8: Appendix H). There were conflicting recommendations for commonly used interventions such as electrotherapy, laser, ultrasound, medication, acupuncture, massage, pulsed electromagnetic therapy, biofeedback and heat/cold. Psychological interventions were recommended by the majority of guidelines (6/7) [23, 26, 46, 61, 64]. The use of soft collars [23, 24, 64] and surgery [23, 25, 63] were not recommended.

### Imaging

Five guidelines referred to imaging for whiplash [23, 25, 45, 63, 64] (Additional file 9: Appendix I). Three out of five of the guidelines referred to using the Canadian Cervical Spine rule to rule out serious pathology following acute whiplash injury [23, 45, 63]. Four guidelines recommended the use of MRI, CT or X-ray for those patients grouped only within a diagnosis category of grade 3 or higher [25, 45, 63, 64]. Routine imaging was not recommended by all guidelines.

## Invasive techniques

Ten guidelines were deemed eligible pertaining to interventional techniques (Table 3). These included invasive interventions such as facet joint injections, nerve blocks, neuro-augmentation (spinal cord stimulation and peripheral nerve stimulation), endoscopic discectomy and implantable drug delivery systems. Guideline publication dates spanned from 2005 to 2013. All but one guideline focused upon chronic neck pain [28]. However, no guideline provided definitions for chronic neck pain (type or chronicity). All the guidelines were authored by physician groups. Three lead authors authored all the included interventional pain guidelines (Easa [28]; Boswell [47, 48]; Manchikanti [19, 49, 50, 66–69]). All failed to outline barriers and facilitators to implementation, strategies to improve uptake and resource implications of applying the guideline with the exception of one [19]. Overall scores for the guidelines did not improved since 2004. The highest quality guidelines were those of Manchikanti, followed by Easa [28] and Boswell [47].

**Table 3:** AGREE II Scores for Invasive Technique Guidelines

Author	Year	Scope and Purpose	Stakeholder involvement	Rigor of Development	Clarity of Presentation	Applicability	Editorial Independence	Overall Score
Boswell [47]	2005	65.3	58.3	42.2	51.4	26.0	56.3	4
Boswell [48]	2007	72.2	77.8	30.6	33.3	40.3	55.6	4
Manchikanti, {Reassessment of Evidence Synthesis} [67]	2008	68.5	33.3	60.4	57.4	18.1	61.1	4
Manchikanti, {Evidence-Based Guidelines} [68]	2009	77.8	59.7	35.9	63.9	37.5	72.9	5
Manchikanti, {Review of neurophysiologic basis} [49]	2009	47.2	30.6	36.5	65.3	15.6	58.3	3
Manchikanti, {Review of therapeutic interventions} [66]	2009	43.1	26.4	34.4	51.4	16.7	60.4	3
Manchikanti, {An algorithmic approach} [50]	2009	53.7	35.2	30.6	77.8	40.3	55.6	4
Manchikanti, {An introduction to an evidence-based approach} [19]	2009	88.9	44.4	52.1	52.8	54.2	87.5	5
Easa [28]	2011	81.5	35.2	54.9	46.3	13.9	44.4	4
Manchikanti [69]	2013	77.8	66.7	64.6	77.8	29.2	83.3	5

### Diagnosis

All guidelines for invasive techniques recommended similar interventions for diagnosis [19, 28, 47, 49] and these included transforaminal epidural steroid injections, selective nerve root blocks and facet joint nerve blocks for those with chronic NP (Additional file 10: Appendix J).

### Prognosis

All guidelines in this group did not formally cover prognostic indicators. Only one guideline recommended age > 50 being a factor for a positive outcome with transforaminal epidural steroid injections [28].

### Intervention

Therapeutically, all invasive technique guidelines recommended epidural steroid injections, medial branch blocks and percutaneous adhesiolysis for chronic NP. The use of Implantable intrathecal systems was originally supported [19, 47]. However, Manchikanti highlights the limited available evidence surrounding its efficacy in managing pain. Radiofrequency neurotomy used for chronic neck pain is recommended by all guidelines except Easa’s [28] that did not cover this treatment modality. Boswell’s guidelines [47] specifically recommend medial branch neurotomy but also deemed intraarticular facet joint injections to be ineffective (Machikanti similarly mentioned limited evidence supporting its use).

Notable differences existed between the recommendations of interventional-focused guidelines and general NP guidelines that have an interventional section. While interventional pain guidelines strongly supported the use of percutaneous adhesiolysis, two other physician authored guidelines by the Colorado Division of Workers [42] and the New York Worker’s Compensation Board [37] recommended against its use. These latter mentioned guidelines along with three others [23, 63] did not recommend steroid injections, despite strong consensus between interventional-focused guidelines on the treatment’s efficacy.

The general NP guidelines, with agreement, recommended against the use of botulinum toxin injections, prolotherapy and disc decompression. Moreover, the guidelines generally advocated the use of surgery in a select or few situations, emphasizing its use only in complex and high-pain cases (Additional file 11: Appendix K).

### Imaging

All the guidelines within this group did not provide an in-depth comparison of various neuroimaging tools such as MRI and CT. However, they did recommend against the use of provocation discography underlined by false-positive rates it tends to produce.

## Neck pain w/associated headache

Four guidelines were included within the diagnosis of headache from 2011 to 2014 (Table 4) [20, 29, 30, 62]. Three out of four guidelines were completed by physician groups [20, 29, 30]. The overall AGREE scores did not change over time. The majority of the guidelines (3/4) failed to outline barriers and facilitators to implementation, strategies to improve uptake and resource implications of applying the guidelines [29, 30, 41].. Two guidelines demonstrated editorial independence being unbiased without competing interests [20, 41]. One guideline was deemed to have a high AGREE II score [20].

**Table 4:** AGREE II Scores for Neck Pain w/Headache Guidelines

Author	Year	Scope and Purpose	Stakeholder involvement	Rigor of Development	Clarity of Presentation	Applicability	Editorial Independence	Overall Score
*Sandrini* [29]	2011	50.0	41.7	43.8	75.0	10.4	16.7	4
*Beithon* [20]	2013	88.9	75.0	64.6	75.0	68.8	95.8	6
*Douglas* [30]	2014	47.2	52.8	50.0	61.1	20.8	4.2	4
*Bryans* [62]	2014	61.1	61.1	65.5	66.7	31.3	66.7	5

### Diagnosis

Guidelines in this category did not routinely distinguish between primary and secondary headache. For the purpose of this paper we only extracted information for those headache types related to NP disorders and this included the focus of recommendations taken in regard to non-acute, non-traumatic and cervicogenic (secondary) headache. Sandirini [29] recommended manual palpation as the most specific and sensitive test (in comparison to EMG and pain pressure threshold) to diagnose headaches types. Bryans [62] recommended the use of the International Classification of Headache disorders 2 (International Headache society-IHS) to categorize headache diagnosis. Beithon [20] suggested a detailed history and examination focused on both physical and neurological examination for differentiation. Diagnosis and differentiation of groups was made mostly by history taking and symptom identification [20, 29, 41].

### Intervention

Two guidelines outlined intervention for cervicogenic headache [45, 62]. Bryans [62] recommends spinal manipulation for cervicogenic headache. Joint mobilization and deep neck flexor exercises were also recommended without the use of them in combination. (Additional file 8: Appendix H). In contrast, Blanpied [45] does not recommend spinal manipulation for acute and chronic NP with headache but only for subacute NP with headache. Active mobility exercise and SNAG exercises were also recommended for acute and subacute categories.

### Imaging

Two CPG’s outlined imaging guidelines for headaches [29, 30] (Additional file 9: Appendix I). Both guidelines agreed that for adults with non-acute headache with no change in symptoms or neurological symptoms, routine imaging was not warranted. Other tests outlined in both guidelines with poor diagnostic value included MRI, CT, EMG, Electroencephalography (EEG), Single-photon emission computed tomography (SPECT scan) and Trans cranial Doppler.

## Cervical arterial dysfunction (cad)

Five guidelines were included that pertained to Cervical Arterial Dysfunction (CAD) [31, 32, 34, 35, 70] (Table 4). Publication dates spanned 10 years (2004–2014). Physiotherapist and chiropractor groups that focused upon diagnosis and pre-manipulative testing protocols authored four of the five guidelines [31, 32, 34, 35]. The remaining guideline focused upon diagnostic imaging and intervention which was authored by a physician group [70]. Four out of the five guidelines did not demonstrate editorial independence [31, 32, 34, 35]. All guidelines failed to identify barriers and facilitators to implementation, strategies to improve uptake and outline resource implication of its application. One high AGREE II scoring guideline was identified within this group [70].

### Diagnosis

Four out of five of the guidelines main objectives were for early diagnosis and presentation of Cervical Arterial Dysfunction [31, 32, 34, 35]. Symptoms presented were gathered with risk factors/prognostic factors prior to cervical manipulation or intervention. Much of the diagnosis procedures were focused on the history of the presentation and the identification of symptoms. Rivett [32] reported that dizziness was the most common symptom reported in CAD. A list of different symptoms reported in the guidelines and are detailed in Additional file 11: Appendix K. Furthermore, three guidelines indicated that pre-manipulative testing/end range rotation/quadrant testing was beneficial in determining CAD symptoms [31, 32, 35]. Cervical instability tests as well as palpation lacked evidence to be supported by any guideline but was recommended in the most recent publication [35].

### Risk factors

None of the guidelines detailed prognostic factor associated with poor prognosis. Rather, risk factors were outlined for Cervical Arterial Dysfunction. Rushton [35] indicates that hypertension and cervical instability are two of the largest risk factors to VAI. Anderson and Peacock state absolute risk factors for manipulation are 1) Signs of neurovascular impairment 2) Sharp neck or occipital pain; 3) Severe and persistent headache that is unlike another. Three guidelines indicated pain/headache (unlike any other they have ever experience) was a strong risk factor for establishing CAD [32, 34, 35]. A list of absolute risk factors for manipulation from all the guidelines can be found here (Additional file 12: Appendix L).

### Intervention

Harrigan [70] was the only guideline that presented recommendations regarding interventions for Cervical Arterial Dysfunction. It was recommended that no conclusive evidence supports treatment for CAD, but most clinicians support the use of either anti-coagulation or anti-platelet therapy. Due to the inherent risk of haemorrhagic complications from anti-coagulation therapy, it is not considered in multiple trauma patients. Anti-platelet therapy (aspirin) was presented as a safe and comparable option. This guideline was also the highest scoring within the group and had a high overall score.

### Diagnostic imaging

Harrigan [70] recommended imaging techniques for Cervical Arterial Dysfunction (Additional file 9: Appendix I). Catheter Angiography was listed as the gold standard for diagnosis. Alternatively, magnetic resonance angiography (MRA) or computerized tomography angiography (CTA) were presented as quicker and non-invasive options for diagnosis. Duplex sonography was a less common procedure but also effective.

## Discussion

This review of 46 CPG’s found substantial limitations in AGREE II scores pertaining to mechanical NP. In addition, significant heterogeneity was presented in regard to their recommendations. Previous reviews for NP guidelines have concluded that guidelines were overall poor quality and lacked methodological consistency [7, 8]. Our results have agreed with these findings albeit they are improving in more recent years for some NP sub-types. In particular, most guidelines were found to have major limitations with systematic search methods used for evidence, criteria of selecting evidence and adequate descriptions of the strengths and limitations of the body of evidence. The methods for formulating the recommendations were not often described along with a (lack of) link given to the supporting evidence. Also, few guidelines demonstrated editorial independence from their funding bodies or providing an adequate explanation of their applicability. Scores for newer guidelines were significantly greater than older ones. Recent publications (more predominant after 2012) seem to author their publications using the AGREE as a template where older guidelines did not. The acceptance of the AGREE II as a widely used tool to appraise guidelines could be the reasoning for this in recent years.

### Inconsistent definitions of NP acuity and severity

It was surprising to note the significant heterogeneity that was found for the definitions of NP, whiplash and headache in terms of both durations (acuity) and severity of classification. Due to the lack of appropriate classification standards regarding neck pain the interpretation of their findings as a group must be taken with caution. Although many standardized definitions do exist for diagnosis (ICD-10 codes, etc.) many guidelines have not interpreted and collected evidence based upon these differing definitions. This could also be a reason for the large amount of heterogeneity found in the recommendations for diagnosis, prognostic factors, interventions and imaging. In addition, guidelines failed to identify subgroups likely to benefit from interventions. Newer guidelines were better than older ones however no standardized subgrouping method was presented. Finally, few guidelines mentioned dosing of interventions. Dosing is key component to understanding the effectiveness of interventions and should be included for future research of neck pain trials [71].

### Interventional recommendation

Overall, many of the recommendations for interventions have not changed over time for each of the difference NP disorders considered. Active exercise, manipulation and mobilization are recommended by almost every guideline as the main treatment for NP. Throughout the years the number of trials related to NP have increased and the evidence base has grown substantially. However, no overall changes in recommendations have been observed. The interventional guidelines have also followed this trend. If anything, some recommendations have been weakened with the emergence of evidence showing lacking efficacy among treatment modalities, such as was the case with implantable intrathecal infusion systems.

When grading the evidence throughout the publications, many had few “grade A” recommendations and used low quality evidence and consensus to determine their recommendations. Although the amount of evidence may have increased it appears the quality of these that the guidelines are based upon has not.

### Strengths and limitations of the review findings

While many guidelines intertwine recommendations for NP with other areas of pain (such as the lower back), it may be difficult and time-consuming for clinicians to extract the relevant information that could be implemented in their own practice. This review resolves this by filtering the pertinent information from each guideline and summarizing key similarities and differences in simple charts. Furthermore, a clinician who independently reads one of the guidelines included in this systematic review would not have a basis of comparison with regards to methodology and compilation of evidence, facing the risk of blindly accepting recommendations without having assessment of quality. This review provided a detailed assessment and comparison for clinicians that are based upon explicit and transparent observations between all available literatures. The use of the widely recognized AGREE II instrument not only provides an objective way of evaluating the quality of guidelines, but also standardizes the assigned scores. Ideally, anyone can view each domain’s criteria and know precisely why the guideline in question received the score that it did. Also, guidelines were reviewed by at least two and up to four investigators, reducing potential deviations due to personal subjectivity. Most guidelines were scored very similarly between investigators, suggesting high inter-rater reliability.

It is evident that the classification of NP varied greatly by guideline (Additional file 5: Appendix E), with some guidelines failing to produce any distinction between chronic and acute forms of NP. Other differences in recommendations include ambiguity in recommended dosing and local variations in treatment techniques. All these differences render it challenging to formulate a summary of NP recommendations that will be applicable for all categories of patients. Similarly, many guidelines graded their evidence differently assigning unique meaning to phrases such as “level II evidence or grade A”. As such, in order to compare and contrast the guidelines these needed to be ignored and the recommendations were valued as either favoring (+), not favoring (−) or insufficient (I).

Despite great efforts to find and include all relevant NP guidelines, there is a possibility that some guidelines may have been missed by the literature search. Although our search was comprehensive there is always a possibility of some guidelines that were not identified, and this limitation is a feature of all systematic reviews. We did only include CPG’s published in English and therefore there may have been some relevant CPGs excluded.

### Recommendations for future guideline development

It is recommended that a standardized classification system be employed when defining NP, and that guidelines create recommendations tailored to certain subgroups. For example, guidelines should clearly distinguish between recommendations for a younger individual with acute NP that is minimal or an elder who has had intense pain for over 5 years. Other improvements in clarity can be made with regards to the recommendations themselves. For instance, guidelines often recommend medication like epidural steroid injections or patient education but fail to clarify effective dose intervals or what constitutes the umbrella term ‘education’. Uncertainties among clinicians could not only delay patient recovery, but also exacerbate the condition. Some guidelines pertaining to headache did not distinguish between primary and secondary headache. Future guidelines must not only distinguish between these categories but also give recommendations individually based upon these distinctions. Finally, it is recommended that guideline development groups are comprised of diverse stakeholders that include both professionals and patients, to allow for a more representative scope of NP. Then, the conflicting differences between what physiotherapists, physicians, massage therapists, chiropractors and other professionals are minimized. Only three guidelines (of the 46) obtained the views of patients when developing recommendations [52, 54, 56]. This remains a very large limitation for all guidelines and their development processes.

Most clinical guidelines included within this review did not identify or discuss factors that may facilitate or create barriers for their dissemination and adoption nor have authors given recommendations on how to implement them into clinical practice. These guidelines have translated complex evidence into pragmatic recommendations that are simplified to enhance practice patterns or to be so generic in format that the clinician has no clear direction. In our judgement the majority of guideline developers did not seem to explicitly state how their recommendations would be translated into policy or practice even though these were likely discussed and considered during the development process. This may reflect either a lack of knowledge on how to promote adaptation and implementation or that they felt this was beyond the scope of the guideline developmental process. Determinants are factors that obstruct or enable changes in target professional behaviours or the healthcare delivery process [72]. Increasingly, research on guideline development has been focused on identifying guideline determinants such as frameworks [73], taxonomies [74, 75] and checklists [76] in order to improve their utilization within professional practice. However, further research is needed to develop objective mechanisms by which to choose implementation strategies that match the identified barriers [77]. In the future, guideline developers must consider these determinants for uptake and implementation and by doing so serve to partner to overcome potential barriers; otherwise explicit discussion about how the guideline developers consider these factors would allow them to partner and facilitate strategies to overcome these barriers.

Although many clinical trials are conducted yearly pertaining to mechanical NP, the recommended treatments have not changed much in the past decade. This raises doubts about continuous resource allocation to the testing of the same treatment modalities, when the outcomes are reinforcing the same doubt that guidelines have on the efficacy of many of these treatments.

## Conclusion

Most guidelines related to mechanical NP are of poor quality as assessed by the AGREE II but those published from 2012 are rated of higher quality in all domains. Despite an increase in the evidence base, treatment recommendations have not changed significantly over time in their recommendations for interventions used to manage NP.

## Additional files

Additional file 1: **Appendix A** Search terms used within MEDLINE and all search engines for all areas in the overview of reviews across different clinical areas for the management of NP (DOCX 50 kb)

Additional file 2: **Appendix B** PRISMA Flow diagram (DOCX 68 kb)

Additional file 3: **Appendix C** Demographics table for all guidelines reviewed (DOCX 17 kb)

Additional file 4: **Appendix D** Combined table for all AGREE scores for all guidelines reviewed (DOCX 21 kb)

Additional file 5: **Appendix E** Combined table for diagnostic recommendations (Outcome measures, Neck pain classification and clinical tests) for general neck pain and whiplash guidelines. (DOCX 17 kb)

Additional file 6: **Appendix F** Combined table for prognostics factors for general neck pain and whiplash guidelines (DOCX 20 kb)

Additional file 7: **Appendix G** Combined table for psychological prognostic factors for neck pain and whiplash guidelines (DOCX 19 kb)

Additional file 8: **Appendix H** Combined table for interventional/treatment recommendations for general neck pain and whiplash guidelines (DOCX 30 kb)

Additional file 9: **Appendix I** Imaging. Combined table for imaging recommendations for all guidelines (DOCX 17 kb)

Additional file 10: **Appendix J** Combined table for diagnostic recommendations among interventional focused guidelines (DOCX 14 kb)

Additional file 11: **Appendix K** Combined table for all invasive techniques recommended by interventional and non-interventional focused guidelines (DOCX 20 kb)

Additional file 12: **Appendix L** Combined table for VAI risk factors and testing procedures (DOCX 14 kb)

## Abbreviations

CPG: Clinical Practice Guideline
CTA: Computerized Tomography Angiography
MRA: Magnetic Resonance Angiography
NP: Neck Pain
SR: Systematic Review
VAI: Vertebral Artery Insufficiency
