# Supplementary material for: Comparison of CPG’s for the diagnosis, prognosis and management of non-specific neck pain: a systematic review
Source: BMC Musculoskelet Disord. 2019 Feb 14;20:81. doi: 10.1186/s12891-019-2441-3 (PMC6376764; doi:10.1186/s12891-019-2441-3)
Supplement: Supplementary file 1 — Appendix A Search terms used within MEDLINE and all search engines for all areas in the overview of reviews across different clinical areas for the management of NP (DOCX 50 kb) [file 12891_2019_2441_MOESM1_ESM.docx]

Additional file 1: **Appendix A** Search terms used within MEDLINE for all areas in the overview of reviews across different clinical areas for the management of NP

**ICON-Diagnosis**

Medline-OVID

Sept 13 2010

1. Neck Pain/

2. exp Brachial Plexus Neuropathies/

3. exp neck injuries/ or exp whiplash injuries/

4. cervical pain.mp.

5. neckache.mp.

6. whiplash.mp.

7. cervicodynia.mp.

8. cervicalgia.mp.

9. brachialgia.mp.

10. brachial neuritis.mp.

11. brachial neuralgia.mp.

12. neck pain.mp.

13. neck injur*.mp.

14. brachial plexus neuropath*.mp.

15. brachial plexus neuritis.mp.

16. thoracic outlet syndrome/ or cervical rib syndrome/

17. Torticollis/

18. exp brachial plexus neuropathies/ or exp brachial plexus neuritis/

19. cervico brachial neuralgia.ti,ab.

20. cervicobrachial neuralgia.ti,ab.

21. (monoradicul* or monoradicl*).tw.

22. or/1-21

23. exp headache/ and cervic*.tw.

24. exp genital diseases, female/

25. genital disease*.mp.

26. or/24-25

27. 23 not 26

28. 22 or 27

29. neck/

30. neck muscles/

31. exp cervical plexus/

32. exp cervical vertebrae/

33. atlanto-axial joint/

34. atlanto-occipital joint/

35. Cervical Atlas/

36. spinal nerve roots/

37. exp brachial plexus/

38. (odontoid* or cervical or occip* or atlant*).tw.

39. axis/ or odontoid process/

40. Thoracic Vertebrae/

41. cervical vertebrae.mp.

42. cervical plexus.mp.

43. cervical spine.mp.

44. (neck adj3 muscles).mp.

45. (brachial adj3 plexus).mp.

46. (thoracic adj3 vertebrae).mp.

47. neck.mp.

48. (thoracic adj3 spine).mp.

49. (thoracic adj3 outlet).mp.

50. trapezius.mp.

51. cervical.mp.

52. cervico*.mp.

53. 51 or 52

54. exp genital diseases, female/

55. genital disease*.mp.

56. exp *Uterus/

57. 54 or 55 or 56

58. 53 not 57

59. 29 or 30 or 31 or 32 or 33 or 34 or 35 or 36 or 37 or 38 or 39 or 40 or 41 or 42 or 43 or 44 or 45 or 46 or 47 or 48 or 49 or 50 or 58

60. exp pain/

61. exp injuries/

62. pain.mp.

63. ache.mp.

64. sore.mp.

65. stiff.mp.

66. discomfort.mp.

67. injur*.mp.

68. neuropath*.mp.

69. or/60-68

70. 59 and 69

71. Radiculopathy/

72. exp temporomandibular joint disorders/ or exp temporomandibular joint dysfunction syndrome/

73. myofascial pain syndromes/

74. exp "Sprains and Strains"/

75. exp Spinal Osteophytosis/

76. exp Neuritis/

77. Polyradiculopathy/

78. exp Arthritis/

79. Fibromyalgia/

80. spondylitis/ or discitis/

81. spondylosis/ or spondylolysis/ or spondylolisthesis/

82. radiculopathy.mp.

83. radiculitis.mp.

84. temporomandibular.mp.

85. myofascial pain syndrome*.mp.

86. thoracic outlet syndrome*.mp.

87. spinal osteophytosis.mp.

88. neuritis.mp.

89. spondylosis.mp.

90. spondylitis.mp.

91. spondylolisthesis.mp.

92. or/71-91

93. 59 and 92

94. exp neck/

95. exp cervical vertebrae/

96. Thoracic Vertebrae/

97. neck.mp.

98. (thoracic adj3 vertebrae).mp.

99. cervical.mp.

100. cervico*.mp.

101. 99 or 100

102. exp genital diseases, female/

103. genital disease*.mp.

104. exp *Uterus/

105. or/102-104

106. 101 not 105

107. (thoracic adj3 spine).mp.

108. cervical spine.mp.

109. 94 or 95 or 96 or 97 or 98 or 106 or 107 or 108

110. Intervertebral Disk/

111. (disc or discs).mp.

112. (disk or disks).mp.

113. 110 or 111 or 112

114. 109 and 113

115. herniat*.mp.

116. slipped.mp.

117. prolapse*.mp.

118. displace*.mp.

119. degenerat*.mp.

120. (bulge or bulged or bulging).mp.

121. 115 or 116 or 117 or 118 or 119 or 120

122. 114 and 121

123. intervertebral disk degeneration/ or intervertebral disk displacement/

124. intervertebral disk displacement.mp.

125. intervertebral disc displacement.mp.

126. intervertebral disk degeneration.mp.

127. intervertebral disc degeneration.mp.

128. 123 or 124 or 125 or 126 or 127

129. 109 and 128

130. 28 or 70 or 93 or 122 or 129

131. animals/ not (animals/ and humans/)

132. 130 not 131

133. exp *neoplasms/

134. exp *wounds, penetrating/

135. 133 or 134

136. 132 not 135

137. (sensitiv* or diagnos*).mp. or di.fs.

138. 136 and 137

139. meta-analysis.pt,ti,ab,sh.

140. (meta anal$ or metaanal$).ti,ab,sh.

141. ((methodol$ or systematic$ or quantitativ$) adj3 (review$ or overview$ or survey$)).ti.

142. ((methodol$ or systematic$ or quantitativ$) adj3 (review$ or overview$ or survey$)).ab.

143. ((pool$ or combined or combining) adj (data or trials or studies or results)).ti,ab.

144. (medline or embase or cochrane or pubmed or pub med).ti,ab.

145. or/142-144

146. review.pt,sh.

147. 145 and 146

148. or/139-141

149. 147 or 148

150. guidelines as topic/

151. practice guidelines as topic/

152. guideline.pt.

153. practice guideline.pt.

154. (guideline? or guidance or recommendations).ti.

155. consensus.ti.

156. or/150-155

157. 149 or 156

158. 138 and 157

159. limit 158 to yr="2000 -Current"

**ICON-Outcomes**

Medline-OVID

1. exp "outcome and process assessment (health care)"/ or "outcome assessment (health care)"/ or treatment outcome/

2. outcome?.ti.

3. exp "Range of Motion, Articular"/

4. Pain Measurement/

5. exp disability evaluation/

6. "Recovery of Function"/

7. Questionnaires/

8. self-report.tw.

9. ((impairment or disability or function) adj2 (measure? or scale? or evaluation?)).tw.

10. range of motion.tw.

11. (strength adj2 (measure? or scale? or evaluation?)).tw.

12. (outcome? adj2 (measure* or scale? or indicator?)).tw.

13. or/1-12

14. "reproducibility of results"/

15. exp "Sensitivity and Specificity"/

16. reliability.mp.

17. validity.mp.

18. responsiveness.mp.

19. Psychometrics/

20. rasch.mp.

21. factor analysis, statistical/

22. factor analysis.tw.

23. differential functioning.mp.

24. (validity or validation).mp. [mp=title, original title, abstract, name of substance word, subject heading word, unique identifier]

25. (validity or validation).mp.

26. item difficulty.mp.

27. translation.tw.

28. or/14-27

29. 13 and 28

30. Neck Pain/

31. exp Brachial Plexus Neuropathies/

32. exp neck injuries/ or exp whiplash injuries/

33. cervical pain.mp.

34. neckache.mp.

35. whiplash.mp.

36. cervicodynia.mp.

37. cervicalgia.mp.

38. brachialgia.mp.

39. brachial neuritis.mp.

40. brachial neuralgia.mp.

41. neck pain.mp.

42. neck injur*.mp.

43. brachial plexus neuropath*.mp.

44. brachial plexus neuritis.mp.

45. thoracic outlet syndrome/ or cervical rib syndrome/

46. Torticollis/

47. exp brachial plexus neuropathies/ or exp brachial plexus neuritis/

48. cervico brachial neuralgia.ti,ab.

49. cervicobrachial neuralgia.ti,ab.

50. (monoradicul* or monoradicl*).tw.

51. or/30-50

52. exp headache/ and cervic*.tw.

53. exp genital diseases, female/

54. genital disease*.mp.

55. or/53-54

56. 52 not 55

57. 51 or 56

58. neck/

59. neck muscles/

60. exp cervical plexus/

61. exp cervical vertebrae/

62. atlanto-axial joint/

63. atlanto-occipital joint/

64. Cervical Atlas/

65. spinal nerve roots/

66. exp brachial plexus/

67. (odontoid* or cervical or occip* or atlant*).tw.

68. axis/ or odontoid process/

69. Thoracic Vertebrae/

70. cervical vertebrae.mp.

71. cervical plexus.mp.

72. cervical spine.mp.

73. (neck adj3 muscles).mp.

74. (brachial adj3 plexus).mp.

75. (thoracic adj3 vertebrae).mp.

76. neck.mp.

77. (thoracic adj3 spine).mp.

78. (thoracic adj3 outlet).mp.

79. trapezius.mp.

80. cervical.mp.

81. cervico*.mp.

82. 80 or 81

83. exp genital diseases, female/

84. genital disease*.mp.

85. exp *Uterus/

86. 83 or 84 or 85

87. 82 not 86

88. 58 or 59 or 60 or 61 or 62 or 63 or 64 or 65 or 66 or 67 or 68 or 69 or 70 or 71 or 72 or 73 or 74 or 75 or 76 or 77 or 78 or 79 or 87

89. exp pain/

90. exp injuries/

91. pain.mp.

92. ache.mp.

93. sore.mp.

94. stiff.mp.

95. discomfort.mp.

96. injur*.mp.

97. neuropath*.mp.

98. or/89-97

99. 88 and 98

100. Radiculopathy/

101. exp temporomandibular joint disorders/ or exp temporomandibular joint dysfunction syndrome/

102. myofascial pain syndromes/

103. exp "Sprains and Strains"/

104. exp Spinal Osteophytosis/

105. exp Neuritis/

106. Polyradiculopathy/

107. exp Arthritis/

108. Fibromyalgia/

109. spondylitis/ or discitis/

110. spondylosis/ or spondylolysis/ or spondylolisthesis/

111. radiculopathy.mp.

112. radiculitis.mp.

113. temporomandibular.mp.

114. myofascial pain syndrome*.mp.

115. thoracic outlet syndrome*.mp.

116. spinal osteophytosis.mp.

117. neuritis.mp.

118. spondylosis.mp.

119. spondylitis.mp.

120. spondylolisthesis.mp.

121. or/100-120

122. 88 and 121

123. exp neck/

124. exp cervical vertebrae/

125. Thoracic Vertebrae/

126. neck.mp.

127. (thoracic adj3 vertebrae).mp.

128. cervical.mp.

129. cervico*.mp.

130. 128 or 129

131. exp genital diseases, female/

132. genital disease*.mp.

133. exp *Uterus/

134. or/131-133

135. 130 not 134

136. (thoracic adj3 spine).mp.

137. cervical spine.mp.

138. 123 or 124 or 125 or 126 or 127 or 135 or 136 or 137

139. Intervertebral Disk/

140. (disc or discs).mp.

141. (disk or disks).mp.

142. 139 or 140 or 141

143. 138 and 142

144. herniat*.mp.

145. slipped.mp.

146. prolapse*.mp.

147. displace*.mp.

148. degenerat*.mp.

149. (bulge or bulged or bulging).mp.

150. 144 or 145 or 146 or 147 or 148 or 149

151. 143 and 150

152. intervertebral disk degeneration/ or intervertebral disk displacement/

153. intervertebral disk displacement.mp.

154. intervertebral disc displacement.mp.

155. intervertebral disk degeneration.mp.

156. intervertebral disc degeneration.mp.

157. 152 or 153 or 154 or 155 or 156

158. 138 and 157

159. 57 or 99 or 122 or 151 or 158

160. animals/ not (animals/ and humans/)

161. 159 not 160

162. exp *neoplasms/

163. exp *wounds, penetrating/

164. 162 or 163

165. 161 not 164

166. 29 and 165

167. guidelines as topic/

168. practice guidelines as topic/

169. guideline.pt.

170. practice guideline.pt.

171. (guideline? or guidance or recommendations).ti.

172. consensus.ti.

173. or/167-172

174. meta-analysis/

175. exp meta-analysis as topic/

176. (meta analy* or metaanaly* or met analy* or metanaly*).tw.

177. review literature as topic/

178. (collaborative research or collaborative review* or collaborative overview*).tw.

179. (integrative research or integrative review* or intergrative overview*).tw.

180. (quantitative adj3 (research or review* or overview*)).tw.

181. (research integration or research overview*).tw.

182. (systematic* adj3 (review* or overview*)).tw.

183. (methodologic* adj3 (review* or overview*)).tw.

184. exp technology assessment biomedical/

185. (hta or thas or technology assessment*).tw.

186. ((hand adj2 search*) or (manual* adj search*)).tw.

187. ((electronic adj database*) or (bibliographic* adj database*)).tw.

188. ((data adj2 abstract*) or (data adj2 extract*)).tw.

189. (analys* adj3 (pool or pooled or pooling)).tw.

190. mantel haenszel.tw.

191. (cohrane or pubmed or pub med or medline or embase or psycinfo or psyclit or psychinfo or psychlit or cinahl or science citation indes).ab.

192. or/174-191

193. 173 or 192

194. 166 and 193

195. limit 194 to yr="2000 -Current"

**ICON-Prognosis**

Medline-OVID

1. Neck Pain/

2. exp Brachial Plexus Neuropathies/

3. exp neck injuries/ or exp whiplash injuries/

4. cervical pain.mp.

5. neckache.mp.

6. whiplash.mp.

7. cervicodynia.mp.

8. cervicalgia.mp.

9. brachialgia.mp.

10. brachial neuritis.mp.

11. brachial neuralgia.mp.

12. neck pain.mp.

13. neck injur*.mp.

14. brachial plexus neuropath*.mp.

15. brachial plexus neuritis.mp.

16. thoracic outlet syndrome/ or cervical rib syndrome/

17. Torticollis/

18. exp brachial plexus neuropathies/ or exp brachial plexus neuritis/

19. cervico brachial neuralgia.ti,ab.

20. cervicobrachial neuralgia.ti,ab.

21. (monoradicul* or monoradicl*).tw.

22. or/1-21

23. exp headache/ and cervic*.tw.

24. exp genital diseases, female/

25. genital disease*.mp.

26. or/24-25

27. 23 not 26

28. 22 or 27

29. neck/

30. neck muscles/

31. exp cervical plexus/

32. exp cervical vertebrae/

33. atlanto-axial joint/

34. atlanto-occipital joint/

35. Cervical Atlas/

36. spinal nerve roots/

37. exp brachial plexus/

38. (odontoid* or cervical or occip* or atlant*).tw.

39. axis/ or odontoid process/

40. Thoracic Vertebrae/

41. cervical vertebrae.mp.

42. cervical plexus.mp.

43. cervical spine.mp.

44. (neck adj3 muscles).mp.

45. (brachial adj3 plexus).mp.

46. (thoracic adj3 vertebrae).mp.

47. neck.mp.

48. (thoracic adj3 spine).mp.

49. (thoracic adj3 outlet).mp.

50. trapezius.mp.

51. cervical.mp.

52. cervico*.mp.

53. 51 or 52

54. exp genital diseases, female/

55. genital disease*.mp.

56. exp *Uterus/

57. 54 or 55 or 56

58. 53 not 57

59. 29 or 30 or 31 or 32 or 33 or 34 or 35 or 36 or 37 or 38 or 39 or 40 or 41 or 42 or 43 or 44 or 45 or 46 or 47 or 48 or 49 or 50 or 58

60. exp pain/

61. exp injuries/

62. pain.mp.

63. ache.mp.

64. sore.mp.

65. stiff.mp.

66. discomfort.mp.

67. injur*.mp.

68. neuropath*.mp.

69. or/60-68

70. 59 and 69

71. Radiculopathy/

72. exp temporomandibular joint disorders/ or exp temporomandibular joint dysfunction syndrome/

73. myofascial pain syndromes/

74. exp "Sprains and Strains"/

75. exp Spinal Osteophytosis/

76. exp Neuritis/

77. Polyradiculopathy/

78. exp Arthritis/

79. Fibromyalgia/

80. spondylitis/ or discitis/

81. spondylosis/ or spondylolysis/ or spondylolisthesis/

82. radiculopathy.mp.

83. radiculitis.mp.

84. temporomandibular.mp.

85. myofascial pain syndrome*.mp.

86. thoracic outlet syndrome*.mp.

87. spinal osteophytosis.mp.

88. neuritis.mp.

89. spondylosis.mp.

90. spondylitis.mp.

91. spondylolisthesis.mp.

92. or/71-91

93. 59 and 92

94. exp neck/

95. exp cervical vertebrae/

96. Thoracic Vertebrae/

97. neck.mp.

98. (thoracic adj3 vertebrae).mp.

99. cervical.mp.

100. cervico*.mp.

101. 99 or 100

102. exp genital diseases, female/

103. genital disease*.mp.

104. exp *Uterus/

105. or/102-104

106. 101 not 105

107. (thoracic adj3 spine).mp.

108. cervical spine.mp.

109. 94 or 95 or 96 or 97 or 98 or 106 or 107 or 108

110. Intervertebral Disk/

111. (disc or discs).mp.

112. (disk or disks).mp.

113. 110 or 111 or 112

114. 109 and 113

115. herniat*.mp.

116. slipped.mp.

117. prolapse*.mp.

118. displace*.mp.

119. degenerat*.mp.

120. (bulge or bulged or bulging).mp.

121. 115 or 116 or 117 or 118 or 119 or 120

122. 114 and 121

123. intervertebral disk degeneration/ or intervertebral disk displacement/

124. intervertebral disk displacement.mp.

125. intervertebral disc displacement.mp.

126. intervertebral disk degeneration.mp.

127. intervertebral disc degeneration.mp.

128. 123 or 124 or 125 or 126 or 127

129. 109 and 128

130. 28 or 70 or 93 or 122 or 129

131. animals/ not (animals/ and humans/)

132. 130 not 131

133. exp *neoplasms/

134. exp *wounds, penetrating/

135. 133 or 134

136. 132 not 135

137. incidence/ or exp mortality/

138. follow-up studies/

139. prognos*.tw.

140. predict*.tw.

141. course*.tw.

142. or/137-141

143. 136 and 142

144. animals/ not (animals/ and humans/)

145. 143 not 144

146. meta-analysis/

147. exp meta-analysis as topic/

148. (meta analy* or metaanaly* or met analy* or metanaly*).tw.

149. review literature as topic/

150. (collaborative research or collaborative review* or collaborative overview*).tw.

151. (integrative research or integrative review* or intergrative overview*).tw.

152. (quantitative adj3 (research or review* or overview*)).tw.

153. (research integration or research overview*).tw.

154. (systematic* adj3 (review* or overview*)).tw.

155. (methodologic* adj3 (review* or overview*)).tw.

156. exp technology assessment biomedical/

157. (hta or thas or technology assessment*).tw.

158. ((hand adj2 search*) or (manual* adj search*)).tw.

159. ((electronic adj database*) or (bibliographic* adj database*)).tw.

160. ((data adj2 abstract*) or (data adj2 extract*)).tw.

161. (analys* adj3 (pool or pooled or pooling)).tw.

162. mantel haenszel.tw.

163. (cohrane or pubmed or pub med or medline or embase or psycinfo or psyclit or psychinfo or psychlit or cinahl or science citation indes).ab.

164. or/146-163

165. 145 and 164

166. guidelines as topic/

167. practice guidelines as topic/

168. guideline.pt.

169. practice guideline.pt.

170. (guideline? or guidance or recommendations).ti.

171. consensus.ti.

172. or/166-171

173. 145 and 172

174. 165 or 173

175. limit 174 to yr="2000 -Current"

ICON Treatment Detailed Search Strategies for MEDLINE

**ICON_Physical Medicine_Treatment**

Medline-OVID

1. Neck Pain/

2. exp Brachial Plexus Neuropathies/

3. exp neck injuries/ or exp whiplash injuries/

4. cervical pain.mp.

5. neckache.mp.

6. whiplash.mp.

7. cervicodynia.mp.

8. cervicalgia.mp.

9. brachialgia.mp.

10. brachial neuritis.mp.

11. brachial neuralgia.mp.

12. neck pain.mp.

13. neck injur*.mp.

14. brachial plexus neuropath*.mp.

15. brachial plexus neuritis.mp.

16. thoracic outlet syndrome/ or cervical rib syndrome/

17. Torticollis/

18. exp brachial plexus neuropathies/ or exp brachial plexus neuritis/

19. cervico brachial neuralgia.ti,ab.

20. cervicobrachial neuralgia.ti,ab.

21. (monoradicul* or monoradicl*).tw.

22. or/1-21

23. exp headache/ and cervic*.tw.

24. exp genital diseases, female/

25. genital disease*.mp.

26. or/24-25

27. 23 not 26

28. 22 or 27

29. neck/

30. neck muscles/

31. exp cervical plexus/

32. exp cervical vertebrae/

33. atlanto-axial joint/

34. atlanto-occipital joint/

35. Cervical Atlas/

36. spinal nerve roots/

37. exp brachial plexus/

38. (odontoid* or cervical or occip* or atlant*).tw.

39. axis/ or odontoid process/

40. Thoracic Vertebrae/

41. cervical vertebrae.mp.

42. cervical plexus.mp.

43. cervical spine.mp.

44. (neck adj3 muscles).mp.

45. (brachial adj3 plexus).mp.

46. (thoracic adj3 vertebrae).mp.

47. neck.mp.

48. (thoracic adj3 spine).mp.

49. (thoracic adj3 outlet).mp.

50. trapezius.mp.

51. cervical.mp.

52. cervico*.mp.

53. 51 or 52

54. exp genital diseases, female/

55. genital disease*.mp.

56. exp *Uterus/

57. 54 or 55 or 56

58. 53 not 57

59. 29 or 30 or 31 or 32 or 33 or 34 or 35 or 36 or 37 or 38 or 39 or 40 or 41 or 42 or 43 or 44 or 45 or 46 or 47 or 48 or 49 or 50 or 58

60. exp pain/

61. exp injuries/

62. pain.mp.

63. ache.mp.

64. sore.mp.

65. stiff.mp.

66. discomfort.mp.

67. injur*.mp.

68. neuropath*.mp.

69. or/60-68

70. 59 and 69

71. Radiculopathy/

72. exp temporomandibular joint disorders/ or exp temporomandibular joint dysfunction syndrome/

73. myofascial pain syndromes/

74. exp "Sprains and Strains"/

75. exp Spinal Osteophytosis/

76. exp Neuritis/

77. Polyradiculopathy/

78. exp Arthritis/

79. Fibromyalgia/

80. spondylitis/ or discitis/

81. spondylosis/ or spondylolysis/ or spondylolisthesis/

82. radiculopathy.mp.

83. radiculitis.mp.

84. temporomandibular.mp.

85. myofascial pain syndrome*.mp.

86. thoracic outlet syndrome*.mp.

87. spinal osteophytosis.mp.

88. neuritis.mp.

89. spondylosis.mp.

90. spondylitis.mp.

91. spondylolisthesis.mp.

92. or/71-91

93. 59 and 92

94. exp neck/

95. exp cervical vertebrae/

96. Thoracic Vertebrae/

97. neck.mp.

98. (thoracic adj3 vertebrae).mp.

99. cervical.mp.

100. cervico*.mp.

101. 99 or 100

102. exp genital diseases, female/

103. genital disease*.mp.

104. exp *Uterus/

105. or/102-104

106. 101 not 105

107. (thoracic adj3 spine).mp.

108. cervical spine.mp.

109. 94 or 95 or 96 or 97 or 98 or 106 or 107 or 108

110. Intervertebral Disk/

111. (disc or discs).mp.

112. (disk or disks).mp.

113. 110 or 111 or 112

114. 109 and 113

115. herniat*.mp.

116. slipped.mp.

117. prolapse*.mp.

118. displace*.mp.

119. degenerat*.mp.

120. (bulge or bulged or bulging).mp.

121. 115 or 116 or 117 or 118 or 119 or 120

122. 114 and 121

123. intervertebral disk degeneration/ or intervertebral disk displacement/

124. intervertebral disk displacement.mp.

125. intervertebral disc displacement.mp.

126. intervertebral disk degeneration.mp.

127. intervertebral disc degeneration.mp.

128. 123 or 124 or 125 or 126 or 127

129. 109 and 128

130. 28 or 70 or 93 or 122 or 129

131. animals/ not (animals/ and humans/)

132. 130 not 131

133. exp *neoplasms/

134. exp *wounds, penetrating/

135. 133 or 134

136. 132 not 135

137. Neck Pain/rh [Rehabilitation]

138. exp Brachial Plexus Neuropathies/rh

139. exp neck injuries/rh or exp whiplash injuries/rh

140. thoracic outlet syndrome/rh or cervical rib syndrome/rh

141. Torticollis/rh

142. exp brachial plexus neuropathies/rh or exp brachial plexus neuritis/rh

143. 137 or 138 or 139 or 140 or 141 or 142

144. Radiculopathy/rh

145. exp temporomandibular joint disorders/rh or exp temporomandibular joint dysfunction syndrome/rh

146. myofascial pain syndromes/rh

147. exp "Sprains and Strains"/rh

148. exp Spinal Osteophytosis/rh

149. exp Neuritis/rh

150. Polyradiculopathy/rh

151. exp Arthritis/rh

152. Fibromyalgia/rh

153. spondylitis/rh or discitis/rh

154. spondylosis/rh or spondylolysis/rh or spondylolisthesis/rh

155. or/144-154

156. 59 and 155

157. exp Combined Modality Therapy/

158. Exercise/

159. Physical Exertion/

160. exp Exercise Therapy/

161. exp Electric Stimulation Therapy/

162. Transcutaneous Electric Nerve Stimulation/

163. pulsed electro magnetic field.mp.

164. pulsed electromagnetic field.tw.

165. Electromagnetic Fields/

166. Magnetic Field Therapy/

167. Electric Stimulation/

168. exp Orthotic Devices/

169. kinesiotaping.tw.

170. taping.tw.

171. oral splints.tw.

172. Occlusal Splints/

173. pillow?.tw.

174. collar?.tw.

175. Traction/

176. traction.tw.

177. exp Laser Therapy/

178. laser therapy.tw.

179. exp Rehabilitation/

180. Ultrasonic Therapy/

181. exp Phototherapy/

182. Lasers/

183. exp Physical Therapy Modalities/

184. repetitive magnetic stimulation.tw.

185. exp Cryotherapy/

186. Hydrotherapy/

187. exp Hyperthermia, Induced/

188. vapocoolant spray.mp.

189. Cryoanesthesia/

190. Ice/

191. postur* correction.mp.

192. Feldenkrais.mp.

193. (alexander adj (technique or method)).tw.

194. Relaxation Therapy/

195. Biofeedback, Psychology/

196. faradic stimulation.mp.

197. or/157-196

198. 136 and 197

199. 143 or 156 or 198

200. animals/ not (animals/ and humans/)

201. 199 not 200

202. guidelines as topic/

203. practice guidelines as topic/

204. guideline.pt.

205. practice guideline.pt.

206. (guideline? or guidance or recommendations).ti.

207. consensus.ti.

208. or/202-207

209. 201 and 208

210. 136 and 208

211. 209 or 210

212. limit 211 to yr="2006 -Current"

213. limit 211 to yr="1902 - 2005"

214. meta-analysis/

215. exp meta-analysis as topic/

216. (meta analy* or metaanaly* or met analy* or metanaly*).tw.

217. review literature as topic/

218. (collaborative research or collaborative review* or collaborative overview*).tw.

219. (integrative research or integrative review* or intergrative overview*).tw.

220. (quantitative adj3 (research or review* or overview*)).tw.

221. (research integration or research overview*).tw.

222. (systematic* adj3 (review* or overview*)).tw.

223. (methodologic* adj3 (review* or overview*)).tw.

224. exp technology assessment biomedical/

225. (hta or thas or technology assessment*).tw.

226. ((hand adj2 search*) or (manual* adj search*)).tw.

227. ((electronic adj database*) or (bibliographic* adj database*)).tw.

228. ((data adj2 abstract*) or (data adj2 extract*)).tw.

229. (analys* adj3 (pool or pooled or pooling)).tw.

230. mantel haenszel.tw.

231. (cohrane or pubmed or pub med or medline or embase or psycinfo or psyclit or psychinfo or psychlit or cinahl or science citation indes).ab.

232. or/214-231

233. 201 and 232

234. limit 233 to yr="2006 -Current"

235. limit 233 to yr="1902 - 2005"

**OR**

**ICON_ManualTherpay_Treatment**

Medline-OVID

1. Neck Pain/

2. exp Brachial Plexus Neuropathies/

3. exp neck injuries/ or exp whiplash injuries/

4. cervical pain.mp.

5. neckache.mp.

6. whiplash.mp.

7. cervicodynia.mp.

8. cervicalgia.mp.

9. brachialgia.mp.

10. brachial neuritis.mp.

11. brachial neuralgia.mp.

12. neck pain.mp.

13. neck injur*.mp.

14. brachial plexus neuropath*.mp.

15. brachial plexus neuritis.mp.

16. thoracic outlet syndrome/ or cervical rib syndrome/

17. Torticollis/

18. exp brachial plexus neuropathies/ or exp brachial plexus neuritis/

19. cervico brachial neuralgia.ti,ab.

20. cervicobrachial neuralgia.ti,ab.

21. (monoradicul* or monoradicl*).tw.

22. or/1-21

23. exp headache/ and cervic*.tw.

24. exp genital diseases, female/

25. genital disease*.mp.

26. or/24-25

27. 23 not 26

28. 22 or 27

29. neck/

30. neck muscles/

31. exp cervical plexus/

32. exp cervical vertebrae/

33. atlanto-axial joint/

34. atlanto-occipital joint/

35. Cervical Atlas/

36. spinal nerve roots/

37. exp brachial plexus/

38. (odontoid* or cervical or occip* or atlant*).tw.

39. axis/ or odontoid process/

40. Thoracic Vertebrae/

41. cervical vertebrae.mp.

42. cervical plexus.mp.

43. cervical spine.mp.

44. (neck adj3 muscles).mp.

45. (brachial adj3 plexus).mp.

46. (thoracic adj3 vertebrae).mp.

47. neck.mp.

48. (thoracic adj3 spine).mp.

49. (thoracic adj3 outlet).mp.

50. trapezius.mp.

51. cervical.mp.

52. cervico*.mp.

53. 51 or 52

54. exp genital diseases, female/

55. genital disease*.mp.

56. exp *Uterus/

57. 54 or 55 or 56

58. 53 not 57

59. 29 or 30 or 31 or 32 or 33 or 34 or 35 or 36 or 37 or 38 or 39 or 40 or 41 or 42 or 43 or 44 or 45 or 46 or 47 or 48 or 49 or 50 or 58

60. exp pain/

61. exp injuries/

62. pain.mp.

63. ache.mp.

64. sore.mp.

65. stiff.mp.

66. discomfort.mp.

67. injur*.mp.

68. neuropath*.mp.

69. or/60-68

70. 59 and 69

71. Radiculopathy/

72. exp temporomandibular joint disorders/ or exp temporomandibular joint dysfunction syndrome/

73. myofascial pain syndromes/

74. exp "Sprains and Strains"/

75. exp Spinal Osteophytosis/

76. exp Neuritis/

77. Polyradiculopathy/

78. exp Arthritis/

79. Fibromyalgia/

80. spondylitis/ or discitis/

81. spondylosis/ or spondylolysis/ or spondylolisthesis/

82. radiculopathy.mp.

83. radiculitis.mp.

84. temporomandibular.mp.

85. myofascial pain syndrome*.mp.

86. thoracic outlet syndrome*.mp.

87. spinal osteophytosis.mp.

88. neuritis.mp.

89. spondylosis.mp.

90. spondylitis.mp.

91. spondylolisthesis.mp.

92. or/71-91

93. 59 and 92

94. exp neck/

95. exp cervical vertebrae/

96. Thoracic Vertebrae/

97. neck.mp.

98. (thoracic adj3 vertebrae).mp.

99. cervical.mp.

100. cervico*.mp.

101. 99 or 100

102. exp genital diseases, female/

103. genital disease*.mp.

104. exp *Uterus/

105. or/102-104

106. 101 not 105

107. (thoracic adj3 spine).mp.

108. cervical spine.mp.

109. 94 or 95 or 96 or 97 or 98 or 106 or 107 or 108

110. Intervertebral Disk/

111. (disc or discs).mp.

112. (disk or disks).mp.

113. 110 or 111 or 112

114. 109 and 113

115. herniat*.mp.

116. slipped.mp.

117. prolapse*.mp.

118. displace*.mp.

119. degenerat*.mp.

120. (bulge or bulged or bulging).mp.

121. 115 or 116 or 117 or 118 or 119 or 120

122. 114 and 121

123. intervertebral disk degeneration/ or intervertebral disk displacement/

124. intervertebral disk displacement.mp.

125. intervertebral disc displacement.mp.

126. intervertebral disk degeneration.mp.

127. intervertebral disc degeneration.mp.

128. 123 or 124 or 125 or 126 or 127

129. 109 and 128

130. 28 or 70 or 93 or 122 or 129

131. animals/ not (animals/ and humans/)

132. 130 not 131

133. exp *neoplasms/

134. exp *wounds, penetrating/

135. 133 or 134

136. 132 not 135

137. Neck Pain/rh, th [Rehabilitation, Therapy]

138. exp Brachial Plexus Neuropathies/rh, th

139. exp neck injuries/rh, th or exp whiplash injuries/rh, th

140. thoracic outlet syndrome/rh, th or cervical rib syndrome/rh, th

141. Torticollis/rh, th

142. exp brachial plexus neuropathies/rh, th or exp brachial plexus neuritis/rh, th

143. or/137-142

144. Radiculopathy/rh, th

145. exp temporomandibular joint disorders/rh, th or exp temporomandibular joint dysfunction syndrome/rh, th

146. myofascial pain syndromes/rh, th

147. exp "Sprains and Strains"/rh, th

148. exp Spinal Osteophytosis/rh, th

149. exp Neuritis/rh, th

150. Polyradiculopathy/rh, th

151. exp Arthritis/rh, th

152. Fibromyalgia/rh, th

153. spondylitis/rh, th or discitis/rh, th

154. spondylosis/rh, th or spondylolysis/rh, th or spondylolisthesis/rh, th

155. or/144-154

156. 59 and 155

157. acupuncture/ or chiropractic/

158. exp Musculoskeletal Manipulations/

159. massage.tw.

160. mobili?ation.tw.

161. Acupuncture Therapy/

162. (acupuncture or acu-puncture or needling or acupressure or mox?bustion).tw.

163. ((neck or spine or spinal or cervical or chiropractic* or musculoskeletal* or musculo-skeletal*) adj3 (adjust* or manipulat* or mobiliz* or mobilis*)).tw.

164. (manual adj therap*).tw.

165. (manipulati* adj (therap* or medicine)).tw.

166. (massag* or reflexolog* or rolfing or zone therap*).tw.

167. Nimmo.mp.

168. exp Vibration/tu [Therapeutic Use]

169. (vibration adj5 (therap* or treatment*)).tw.

170. (Chih Ya or Shiatsu or Shiatzu or Zhi Ya).tw.

171. (flexion adj2 distraction*).tw.

172. (myofascial adj3 (release or therap*)).tw.

173. muscle energy technique*.tw.

174. trigger point.tw.

175. proprioceptive Neuromuscular Facilitation*.tw.

176. cyriax friction.tw.

177. (lomilomi or lomi-lomi or trager).tw.

178. aston patterning.tw.

179. (strain adj counterstrain).tw.

180. (craniosacral therap* or cranio-sacral therap*).tw.

181. (amma or ammo or effleuurage or petrissage or hacking or tapotment).tw.

182. Complementary Therapies/

183. ((complement* or alternat* or osteopthic*) adj (therap* or medicine)).tw.

184. (Tui Na or Tuina).tw.

185. or/157-184

186. 136 and 185

187. 143 or 156 or 186

188. animals/ not (animals/ and humans/)

189. 187 not 188

190. guidelines as topic/

191. practice guidelines as topic/

192. guideline.pt.

193. practice guideline.pt.

194. (guideline? or guidance or recommendations).ti.

195. consensus.ti.

196. or/190-195

197. 189 and 196

198. limit 197 to yr="2006 -Current"

199. limit 197 to yr="1902 -2005"

200. meta-analysis/

201. exp meta-analysis as topic/

202. (meta analy* or metaanaly* or met analy* or metanaly*).tw.

203. review literature as topic/

204. (collaborative research or collaborative review* or collaborative overview*).tw.

205. (integrative research or integrative review* or intergrative overview*).tw.

206. (quantitative adj3 (research or review* or overview*)).tw.

207. (research integration or research overview*).tw.

208. (systematic* adj3 (review* or overview*)).tw.

209. (methodologic* adj3 (review* or overview*)).tw.

210. exp technology assessment biomedical/

211. (hta or thas or technology assessment*).tw.

212. ((hand adj2 search*) or (manual* adj search*)).tw.

213. ((electronic adj database*) or (bibliographic* adj database*)).tw.

214. ((data adj2 abstract*) or (data adj2 extract*)).tw.

215. (analys* adj3 (pool or pooled or pooling)).tw.

216. mantel haenszel.tw.

217. (cohrane or pubmed or pub med or medline or embase or psycinfo or psyclit or psychinfo or psychlit or cinahl or science citation indes).ab.

218. or/200-217

219. 189 and 218

220. limit 219 to yr="2006 -Current"

221. limit 219 to yr="1902 -2005"

**OR**

**ICON_DrugTherapy_Treatment**

Medline-OVID

1. Neck Pain/

2. exp Brachial Plexus Neuropathies/

3. exp neck injuries/ or exp whiplash injuries/

4. cervical pain.mp.

5. neckache.mp.

6. whiplash.mp.

7. cervicodynia.mp.

8. cervicalgia.mp.

9. brachialgia.mp.

10. brachial neuritis.mp.

11. brachial neuralgia.mp.

12. neck pain.mp.

13. neck injur*.mp.

14. brachial plexus neuropath*.mp.

15. brachial plexus neuritis.mp.

16. thoracic outlet syndrome/ or cervical rib syndrome/

17. Torticollis/

18. exp brachial plexus neuropathies/ or exp brachial plexus neuritis/

19. cervico brachial neuralgia.ti,ab.

20. cervicobrachial neuralgia.ti,ab.

21. (monoradicul* or monoradicl*).tw.

22. or/1-21

23. exp headache/ and cervic*.tw.

24. exp genital diseases, female/

25. genital disease*.mp.

26. or/24-25

27. 23 not 26

28. 22 or 27

29. neck/

30. neck muscles/

31. exp cervical plexus/

32. exp cervical vertebrae/

33. atlanto-axial joint/

34. atlanto-occipital joint/

35. Cervical Atlas/

36. spinal nerve roots/

37. exp brachial plexus/

38. (odontoid* or cervical or occip* or atlant*).tw.

39. axis/ or odontoid process/

40. Thoracic Vertebrae/

41. cervical vertebrae.mp.

42. cervical plexus.mp.

43. cervical spine.mp.

44. (neck adj3 muscles).mp.

45. (brachial adj3 plexus).mp.

46. (thoracic adj3 vertebrae).mp.

47. neck.mp.

48. (thoracic adj3 spine).mp.

49. (thoracic adj3 outlet).mp.

50. trapezius.mp.

51. cervical.mp.

52. cervico*.mp.

53. 51 or 52

54. exp genital diseases, female/

55. genital disease*.mp.

56. exp *Uterus/

57. 54 or 55 or 56

58. 53 not 57

59. 29 or 30 or 31 or 32 or 33 or 34 or 35 or 36 or 37 or 38 or 39 or 40 or 41 or 42 or 43 or 44 or 45 or 46 or 47 or 48 or 49 or 50 or 58

60. exp pain/

61. exp injuries/

62. pain.mp.

63. ache.mp.

64. sore.mp.

65. stiff.mp.

66. discomfort.mp.

67. injur*.mp.

68. neuropath*.mp.

69. or/60-68

70. 59 and 69

71. Radiculopathy/

72. exp temporomandibular joint disorders/ or exp temporomandibular joint dysfunction syndrome/

73. myofascial pain syndromes/

74. exp "Sprains and Strains"/

75. exp Spinal Osteophytosis/

76. exp Neuritis/

77. Polyradiculopathy/

78. exp Arthritis/

79. Fibromyalgia/

80. spondylitis/ or discitis/

81. spondylosis/ or spondylolysis/ or spondylolisthesis/

82. radiculopathy.mp.

83. radiculitis.mp.

84. temporomandibular.mp.

85. myofascial pain syndrome*.mp.

86. thoracic outlet syndrome*.mp.

87. spinal osteophytosis.mp.

88. neuritis.mp.

89. spondylosis.mp.

90. spondylitis.mp.

91. spondylolisthesis.mp.

92. or/71-91

93. 59 and 92

94. exp neck/

95. exp cervical vertebrae/

96. Thoracic Vertebrae/

97. neck.mp.

98. (thoracic adj3 vertebrae).mp.

99. cervical.mp.

100. cervico*.mp.

101. 99 or 100

102. exp genital diseases, female/

103. genital disease*.mp.

104. exp *Uterus/

105. or/102-104

106. 101 not 105

107. (thoracic adj3 spine).mp.

108. cervical spine.mp.

109. 94 or 95 or 96 or 97 or 98 or 106 or 107 or 108

110. Intervertebral Disk/

111. (disc or discs).mp.

112. (disk or disks).mp.

113. 110 or 111 or 112

114. 109 and 113

115. herniat*.mp.

116. slipped.mp.

117. prolapse*.mp.

118. displace*.mp.

119. degenerat*.mp.

120. (bulge or bulged or bulging).mp.

121. 115 or 116 or 117 or 118 or 119 or 120

122. 114 and 121

123. intervertebral disk degeneration/ or intervertebral disk displacement/

124. intervertebral disk displacement.mp.

125. intervertebral disc displacement.mp.

126. intervertebral disk degeneration.mp.

127. intervertebral disc degeneration.mp.

128. 123 or 124 or 125 or 126 or 127

129. 109 and 128

130. 28 or 70 or 93 or 122 or 129

131. animals/ not (animals/ and humans/)

132. 130 not 131

133. exp *neoplasms/

134. exp *wounds, penetrating/

135. 133 or 134

136. 132 not 135

137. Neck Pain/dt

138. exp Brachial Plexus Neuropathies/dt

139. exp neck injuries/dt or exp whiplash injuries/dt

140. thoracic outlet syndrome/dt or cervical rib syndrome/dt

141. Torticollis/dt

142. exp brachial plexus neuritis/dt

143. or/137-142

144. Radiculopathy/dt

145. exp temporomandibular joint disorders/dt or exp temporomandibular joint dysfunction syndrome/dt

146. myofascial pain syndromes/dt

147. exp "Sprains and Strains"/dt

148. exp Spinal Osteophytosis/dt

149. exp Neuritis/dt

150. Polyradiculopathy/dt

151. exp Arthritis/dt

152. fibromyalgia/dt

153. spondylitis/dt or discitis/dt

154. spondylosis/dt or spondylolysis/dt or spondylolisthesis/dt

155. or/144-154

156. 59 and 155

157. exp Drug Therapy/

158. exp analgesics/

159. exp anti-inflammatory agents/

160. exp muscle relaxants, central/

161. exp psychotropic drugs/

162. exp neuromuscular agents/

163. exp antidepressive agents/

164. exp tranquilizing agents/

165. exp Botulinum Toxins/

166. botulin*.tw.

167. botox.tw.

168. Prilocaine/

169. exp Nerve Block/

170. Injections, Intra-Articular/

171. injections, intramuscular/ or injections, epidural/ or injections, subcutaneous/ or injections, intradermal/

172. Lidocaine/

173. Morphine/

174. Methylprednisolone/

175. exp Glucocorticoids/

176. or/157-175

177. 136 and 176

178. 143 or 156 or 177

179. guidelines as topic/

180. practice guidelines as topic/

181. guideline.pt.

182. practice guideline.pt.

183. (guideline? or guidance or recommendations).ti.

184. consensus.ti.

185. or/179-184

186. 178 and 185

187. limit 186 to yr="2006 -Current"

188. limit 186 to yr="1902 -2005"

189. meta-analysis/

190. exp meta-analysis as topic/

191. (meta analy* or metaanaly* or met analy* or metanaly*).tw.

192. review literature as topic/

193. (collaborative research or collaborative review* or collaborative overview*).tw.

194. (integrative research or integrative review* or intergrative overview*).tw.

195. (quantitative adj3 (research or review* or overview*)).tw.

196. (research integration or research overview*).tw.

197. (systematic* adj3 (review* or overview*)).tw.

198. (methodologic* adj3 (review* or overview*)).tw.

199. exp technology assessment biomedical/

200. (hta or thas or technology assessment*).tw.

201. ((hand adj2 search*) or (manual* adj search*)).tw.

202. ((electronic adj database*) or (bibliographic* adj database*)).tw.

203. ((data adj2 abstract*) or (data adj2 extract*)).tw.

204. (analys* adj3 (pool or pooled or pooling)).tw.

205. mantel haenszel.tw.

206. (cohrane or pubmed or pub med or medline or embase or psycinfo or psyclit or psychinfo or psychlit or cinahl or science citation indes).ab.

207. or/189-206

208. 178 and 207

209. limit 208 to yr="2006 -Current"

210. limit 208 to yr="1902 -2005"

**ICON-Treatment Patient Education**

Medline-OVID

1. Neck Pain/
2. exp Brachial Plexus Neuropathies/

3 exp neck injuries/ or exp whiplash injuries/

4 cervical pain.mp.

5 neckache.mp.

6 whiplash.mp.

7 cervicodynia.mp.

8 cervicalgia.mp.

9 brachialgia.mp.

10 brachial neuritis.mp.

11 brachial neuralgia.mp.

12 neck pain.mp.

13 neck injur*.mp.

14 brachial plexus neuropath*.mp.

15 brachial plexus neuritis.mp.

16 thoracic outlet syndrome/ or cervical rib syndrome/

17 Torticollis/

18 exp brachial plexus neuropathies/ or exp brachial plexus neuritis/

19 cervico brachial neuralgia.ti,ab.

20 cervicobrachial neuralgia.ti,ab.

21 (monoradicul* or monoradicl*).tw.

22 or/1-21

23 exp headache/ and cervic*.tw.

24 exp genital diseases, female/

25 genital disease*.mp.

26 or/24-25

27 23 not 26

28 22 or 27

29 neck/

30 neck muscles/

31 exp cervical plexus/

32 exp cervical vertebrae/

33 atlanto-axial joint/

34 atlanto-occipital joint/

35 Cervical Atlas/

36 spinal nerve roots/

37 exp brachial plexus/

38 (odontoid* or cervical or occip* or atlant*).tw.

39 axis/ or odontoid process/

40 Thoracic Vertebrae/

41 cervical vertebrae.mp.

42 cervical plexus.mp.

43 cervical spine.mp.

44 (neck adj3 muscles).mp.

45 (brachial adj3 plexus).mp.

46 (thoracic adj3 vertebrae).mp.

47 neck.mp.

48 (thoracic adj3 spine).mp.

49 (thoracic adj3 outlet).mp.

50 trapezius.mp.

51 cervical.mp.

52 cervico*.mp.

53 51 or 52

54 exp genital diseases, female/

55 genital disease*.mp.

56 exp *Uterus/

57 54 or 55 or 56

58 53 not 57

59 29 or 30 or 31 or 32 or 33 or 34 or 35 or 36 or 37 or 38 or 39 or 40 or 41 or 42 or 43 or 44 or 45 or 46 or 47 or 48 or 49 or 50 or 58

60 exp pain/

61 exp injuries/

62 pain.mp.

63 ache.mp.

64 sore.mp.

65 stiff.mp.

66 discomfort.mp.

67 injur*.mp.

68 neuropath*.mp.

69 or/60-68

70 59 and 69

71 Radiculopathy/

72 exp temporomandibular joint disorders/ or exp temporomandibular joint dysfunction syndrome/

73 myofascial pain syndromes/

74 exp "Sprains and Strains"/

75 exp Spinal Osteophytosis/

76 exp Neuritis/

77 Polyradiculopathy/

78 exp Arthritis/

79 Fibromyalgia/

80 spondylitis/ or discitis/

81 spondylosis/ or spondylolysis/ or spondylolisthesis/

82 radiculopathy.mp.

83 radiculitis.mp.

84 temporomandibular.mp.

85 myofascial pain syndrome*.mp.

86 thoracic outlet syndrome*.mp.

87 spinal osteophytosis.mp.

88 neuritis.mp.

89 spondylosis.mp.

90 spondylitis.mp.

91 spondylolisthesis.mp.

92 or/71-91

93 59 and 92

94 exp neck/

95 exp cervical vertebrae/

96 Thoracic Vertebrae/

97 neck.mp.

98 (thoracic adj3 vertebrae).mp.

99 cervical.mp.

100 cervico*.mp.

101 99 or 100

102 exp genital diseases, female/

103 genital disease*.mp.

104 exp *Uterus/

105 or/102-104

106 101 not 105

107 (thoracic adj3 spine).mp.

108 cervical spine.mp. (6506)

109 94 or 95 or 96 or 97 or 98 or 106 or 107 or 108

110 Intervertebral Disk/

111 (disc or discs).mp.

112 (disk or disks).mp.

113 110 or 111 or 112

114 109 and 113

115 herniat*.mp.

116 slipped.mp.

117 prolapse*.mp.

118 displace*.mp.

119 degenerat*.mp.

120 (bulge or bulged or bulging).mp.

121 115 or 116 or 117 or 118 or 119 or 120

122 114 and 121 (2018)

123 intervertebral disk degeneration/ or intervertebral disk displacement/

124 intervertebral disk displacement.mp.

125 intervertebral disc displacement.mp.

126 intervertebral disk degeneration.mp.

127 intervertebral disc degeneration.mp.

128 123 or 124 or 125 or 126 or 127

129 109 and 128

130 28 or 70 or 93 or 122 or 129

131 animals/ not (animals/ and humans/)

132 130 not 131

133 exp *neoplasms/

134 exp *wounds, penetrating/

135 133 or 134

136 132 not 135

137 Patient Education as Topic/

138 exp Professional-Patient Relations/

139 exp Health Education/

140 exp Consumer Satisfaction/

141 Patient Advocacy/

142 Patient Participation/

143 exp Patient Compliance/

144 (professional patient communication or physician patient communication or doctor patient communication or nurse patient communication or dentist patient communication).tw.

145 (professional patient relation: or physician patient relation: or doctor patient relation: or nurse patient relation: or dentist patient relation:).tw.

146 (professional patient interaction: or physician patient interaction: or dentist patient interaction: or chiropractor patient interaction:).tw.

147 (patient physician communication or patient doctor communication or patient nurse communication or patient dentist communication).tw.

148 (patient professional relation: or patient physician relation: or patient doctor relation: or patient nurse relation: or patient dentist relation:).tw.

149 (patient professional interaction: or patient physician interaction: or patient doctor interaction: or patient nurse interaction: or patient dentist interaction:).tw.

150 (educat: adj (patient: or consumer: or health:)).tw.

151 (information adj (patient: or consumer: or health:)).tw.

152 (advice adj (patient: or consumer: or health:)).tw.

153 consumer health information.tw.

154 (shared decisionmaking or informed choice).tw.

155 (shared decision making or informed choice).tw.

156 pamphlets/ or exp teaching materials/

157 Self Care/

158 Information Dissemination/

159 Information Services/

160 Teaching/

161 or/137-160

162 136 and 161

163 exp randomized controlled trials as topic/

164 randomized controlled trial.pt.

165 controlled clinical trial.pt.

166 (random* or sham or placebo*).tw.

167 placebos/

168 random allocation/

169 single blind method/

170 double blind method/

171 ((singl* or doubl* or trebl* or tripl*) adj25 (blind* or dumm* or mask*)).ti,ab.

172 (rct or rcts).tw.

173 (control* adj2 (study or studies or trial*)).tw.

174 or/163-173

175 162 and 174

176 limit 175 to yr="2006 -Current"

177 limit 175 to yr="1902 - 2005"

178 guidelines as topic/

179 practice guidelines as topic/

180 guideline.pt.

181 practice guideline.pt.

182 (guideline? or guidance or recommendations).ti.

183 consensus.ti.

184 or/178-183

185 162 and 184

186 limit 185 to yr="2006 -Current"

187 limit 185 to yr="1902 - 2005"

188 meta-analysis/

189 exp meta-analysis as topic/

190 (meta analy* or metaanaly* or met analy* or metanaly*).tw.

191 review literature as topic/

192 (collaborative research or collaborative review* or collaborative overview*).tw.

193 (integrative research or integrative review* or intergrative overview*).tw.

194 (quantitative adj3 (research or review* or overview*)).tw.

195 (research integration or research overview*).tw.

196 (systematic* adj3 (review* or overview*)).tw.

197 (methodologic* adj3 (review* or overview*)).tw.

198 exp technology assessment biomedical/

199 (hta or thas or technology assessment*).tw.

200 ((hand adj2 search*) or (manual* adj search*)).tw.

201 ((electronic adj database*) or (bibliographic* adj database*)).tw.

202 ((data adj2 abstract*) or (data adj2 extract*)).tw.

203 (analys* adj3 (pool or pooled or pooling)).tw.

204 mantel haenszel.tw.

205 (cohrane or pubmed or pub med or medline or embase or psycinfo or psyclit or psychinfo or psychlit or cinahl or science citation indes).ab.

206 or/188-205

207 162 and 206

208 limit 207 to yr="2006 -Current"

209 limit 207 to yr="1902 - 2005"

210 (ae or to or po or co).fs.

211 (safe or safety or unsafe).tw.

212 (side effect* or side event*).tw.

213 ((adverse or undesirable or harm* or injurious or serious or toxic) adj3 (effect* or event* or reaction* or incident* or outcome*)).tw.

214 (abnormalit* or toxicit* or complication* or consequence* or noxious or tolerabilit*).tw.

215 or/210-214

216 162 and 215

217 limit 216 to yr="2006 -Current"

218 limit 216 to yr="1902 - 2005"

219 limit 185 to yr="2000 -Current"

220 from 219 keep 1-21
