# Supplementary material for: Comparison of CPG’s for the diagnosis, prognosis and management of non-specific neck pain: a systematic review
Source: BMC Musculoskelet Disord. 2019 Feb 14;20:81. doi: 10.1186/s12891-019-2441-3 (PMC6376764; doi:10.1186/s12891-019-2441-3)
Supplement: Supplementary file 2 — Appendix B PRISMA Flow diagram (DOCX 68 kb) [file 12891_2019_2441_MOESM2_ESM.docx]

Additional file 2:

**Appendix B: Flow Diagram**


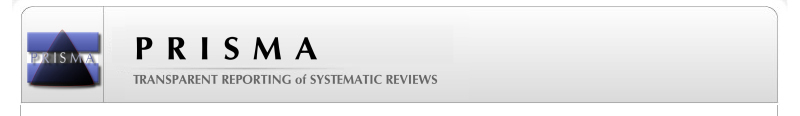
**PRISMA Flow Diagram**

→→→

Full-text articles assessed for eligibility
N = 641

Records excluded
N = 2239

Records screened
N = 2880

Records after duplicates removed
N = 2880

Additional records identified through other sources
N = 20

Records identified through database searching
N = 3082

## Identification

## Screening

Full-text articles excluded,
N = 398

Previous Overview= 394

Not CPG = 4

## Eligibility

Studies included

Previous Overview N = 197

Studies included

Included CPG = 46

## Included
