# Supplementary material for: Comparison of CPG’s for the diagnosis, prognosis and management of non-specific neck pain: a systematic review
Source: BMC Musculoskelet Disord. 2019 Feb 14;20:81. doi: 10.1186/s12891-019-2441-3 (PMC6376764; doi:10.1186/s12891-019-2441-3)
Supplement: Supplementary file 3 — Appendix C Demographics table for all guidelines reviewed (DOCX 17 kb) [file 12891_2019_2441_MOESM3_ESM.docx]

**Additional file 3: APPENDIX C** *DEMOGRAPHICS*

| **Guideline** | **Country** | **Author Panel Composition** | **Intent of Guideline** |
| --- | --- | --- | --- |
| **General Neck Pain** | | | |
| French, 2003 | Australia | Chiro | Img |
| ANAES, 2003 | Australia | PT, MD | Dx, Tx |
| Anderson-Peacock, 2005 | Canada | Chiro, MD | Tx |
| Bussieres, 2008 | Canada | Chiro, RN | Dx, Img |
| New York WC, 2008 | United States | MD | Dx, Img, Tx, Int. |
| Guzman, 2009 | United States | MD, Chiro, PT | Dx, Img, Tx, Int |
| PAC Oklahoma WC, 2009 | United States | MD, Chiro | Dx, Tx, Int. |
| AAMPGG, 2010 | Australia | MD, Chiro, PT, Epidemiologist | Prg, Dx, Tx |
| Bono, 2011 | United States | MD | Dx, Tx, Img |
| Brosseau, 2012 | Canada | MT, MD | Tx |
| Monticone, 2013 | Italy | MD. | Dx, Tx |
| SIGN, 2013 | United Kingdom | MD, PT, RN, OT, Pharm. | Tx |
| Bryans, 2014 | Canada | Chiro,  Occupation Therapy | Tx |
| Colorado Division WC, 2014 | United States | MD | Dx, Img, Tx, Int. |
| Bussieres, 2016 | Canada | Chiro, RN | Tx |
| Cote, 2016 | Canada | Chiro, MD, Rehab Science | Prg, Tx |
| Blanpied, 2017 | United States | PT, MD | Dx, Img, Prg., Tx. |
| Kjaer, 2017 | Denmark | PT, MD | Dx., Tx |
| Bier, 2018 | Netherlands | PT | Dx, Prg, Tx. |
| **Whiplash** | | | |
| Bekkering, 2003 | Netherlands | PT, MD | Dx, Tx, Prg |
| Leigh, 2005 | B.C., Canada | PT | Dx, Tx |
| Mercer, 2007 | London, UK | PT | Tx |
| Trauma Recovery, 2008 | Australia | PT, Chiro, Osteo | Dx, Tx, Prg |
| Davis, 2009 | United States | Chiro | Dx., Tx., Prg., Img. |
| Moore, 2010 | London, UK | PT | Dx, Tx, Prg |
| Bryans, 2010 | Canada | Chiro | Dx, Tx, Prg., Img. |
| MAA WAD, 2014 | New South Wales | MD, PT, Chiro | Dx, Tx, Prg, Img. |
| **Interventional-Focused** | | | |
| Boswell, 2004 | United States | MD | Dx, Int., Img, |
| Boswell, 2007 | United States | MD | Dx, Int., Img |
| Manchikanti, 2008 | United States | MD | Dx, Int, Img, |
| Manchikanti, 2009 [Evidence-Based Guidelines] | United States | MD | Dx. |
| Manchikanti, 2009  [review of neurophysiologic basis] | United States | MD | Dx, Img |
| Manchikanti, 2009  [review of therapeutic interventions] | United States | MD | Dx, Img |
| Manchikanti, 2009  [an algorithmic approach] | United States | MD | Dx, Int, |
| Easa, 2011 | United States | MD | Int |
| Manchikanti, 2013 | United States | MD | Dx, Int, Img |
| **Neck Pain w/Headache** | | | |
| Sandrini, 2011 | Italy  Denmark  The Netherlands  Norway  Spain  Belgium | MD | Dx, Img |
| Beithon, 2013 | United States | MD | Dx, Tx, Img |
| Douglas, 2014 | United States | MD | Dx, Img |
| Bryans, 2014 | Canada | Chiro | Dx |
| **CAD** | | | |
| Magarey, 2004 | Australia | PT | Prg |
| Rivett, 2006 | Australia | PT | Prg |
| Anderson-Peacock, 2007 | Canada | Chiro | Dx, Prg |
| Harrigan, 2013 | United States | MD | Dx, Tx |
| Rushton, 2014 | Australia  Canada  UK  United States | PT | Dx., Prg |

Dx: Diagnosis

Prg: Prognosis

Tx: Treatment

Img: Imaging

Int: Interventional
