# Supplementary material for: Comparison of CPG’s for the diagnosis, prognosis and management of non-specific neck pain: a systematic review
Source: BMC Musculoskelet Disord. 2019 Feb 14;20:81. doi: 10.1186/s12891-019-2441-3 (PMC6376764; doi:10.1186/s12891-019-2441-3)
Supplement: Supplementary file 4 — Appendix D Combined table for all AGREE scores for all guidelines reviewed (DOCX 21 kb) [file 12891_2019_2441_MOESM4_ESM.docx]

Additional file 4: **APPENDIX D** *COMBINED AGREE SCORES FOR ALL GUIDELINES*

| **Author** | **Year** | **Scope and Purpose** | **Stakeholder involvement** | **Rigor of Development** | **Clarity of Presentation** | **Applicability** | **Editorial Independence** | **Overall Score** |
| --- | --- | --- | --- | --- | --- | --- | --- | --- |
| **General Neck Pain** | | | | | | | | |
| French | 2003 | 53.7 | 53.7 | 16.7 | 44.4 | 22.2 | 2.8 | 2 |
|  | 2003 | 50.0 | 52.8 | 19.8 | 36.1 | 22.9 | 8.3 | 2 |
| Anderson-Peacock | 2005 | 79.6 | 63.0 | 58.3 | 79.6 | 40.3 | 47.2 | 5 |
| Bussieres | 2008 | 72.2 | 73.6 | 40.6 | 61.1 | 32.3 | 72.9 | 4 |
| Guzman | 2008 | 72.2 | 83.3 | 67.7 | 77.8 | 39.6 | 91.7 | 6 |
| Physician Advisory Committee | 2009 | 19.4 | 22.2 | 15.6 | 83.3 | 20.8 | 12.5 | 1 |
| Australian Acute Musculoskeletal Pain Guidelines Group | 2010 | 64.8 | 75.9 | 53.5 | 81.5 | 31.9 | 58.3 | 5 |
| New York State Workers | 2010 | 58.3 | 25.0 | 23.7 | 63.9 | 4.2 | 4.2 | 2 |
| Bono | 2011 | 77.8 | 52.8 | 40.6 | 58.3 | 29.2 | 79.2 | 4 |
| Brosseau | 2012 | 61.1 | 59.3 | 55.6 | 48.1 | 2.8 | 19.4 | 4 |
| Monticone | 2013 | 50.0 | 38.9 | 44.8 | 58.3 | 16.7 | 8.3 | 3 |
| SIGN | 2013 | 66.7 | 77.8 | 74.0 | 77.8 | 68.8 | 54.2 | 6 |
| Newman | 2013 | 61.1 | 50.0 | 58.3 | 75.0 | 35.4 | 16.7 | 4 |
| Bryans | 2014 | 69.4 | 33.3 | 50.0 | 66.7 | 20.8 | 37.5 | 3 |
| Colorado Division of Workers | 2014 | 61.1 | 41.7 | 19.8 | 83.3 | 35.4 | 8.3 | 3 |
| Bussieres | 2016 | 88.9 | 55.6 | 66.7 | 72.2 | 62.5 | 79.2 | 5 |
| Cote | 2016 | 83.3 | 86.1 | 75.0 | 80.6 | 52.1 | 91.7 | 7 |
| Blanpied | 2017 | 77.8 | 52.8 | 40.6 | 58.3 | 29.2 | 79.2 | 6 |
| Kjaer | 2017 | 97.2 | 86.1 | 72.9 | 83.3 | 18.8 | 87.5 | 7 |
| Bier | 2018 | 72.2 | 55.6 | 41.7 | 77.8 | 25.0 | 66.7 | 4 |
| **Whiplash** | | | | | | | | |
| Leigh | 2005 | 55.6 | 36.1 | 37.5 | 36.1 | 18.8 | 58.3 | 3 |
| Mercer | 2007 | 80.6 | 75.0 | 62.5 | 44.4 | 25.0 | 8.3 | 5 |
| TRACsa | 2008 | 75.0 | 72.2 | 61.5 | 80.6 | 37.5 | 8.3 | 6 |
| Davis | 2009 | 55.6 | 30.6 | 20.8 | 41.7 | 14.6 | 8.3 | 2 |
| Bryans | 2010 | 75.0 | 55.6 | 33.3 | 47.2 | 35.4 | 12.5 | 3 |
| Moore | 2010 | 94.4 | 77.8 | 92.7 | 80.6 | 85.4 | 70.8 | 7 |
| MAA | 2014 | 86.1 | 69.4 | 54.2 | 88.9 | 22.9 | 20.8 | 4 |
| **Interventional-Focused** | | | | | | | | |
| Boswell | 2005 | 65.3 | 58.3 | 42.2 | 51.4 | 26.0 | 56.3 | 4 |
| Boswell | 2007 | 72.2 | 77.8 | 30.6 | 33.3 | 40.3 | 55.6 | 4 |
| Manchikanti, {Reassessment of Evidence Synthesis} | 2008 | 68.5 | 33.3 | 60.4 | 57.4 | 18.1 | 61.1 | 4 |
| Manchikanti, {Evidence-Based Guidelines} | 2009 | 77.8 | 59.7 | 35.9 | 63.9 | 37.5 | 72.9 | 5 |
| Manchikanti,  {Review of neurophysiologic basis} | 2009 | 47.2 | 30.6 | 36.5 | 65.3 | 15.6 | 58.3 | 3 |
| Manchikanti, {Review of therapeutic interventions} | 2009 | 43.1 | 26.4 | 34.4 | 51.4 | 16.7 | 60.4 | 3 |
| Manchikanti,  {An algorithmic approach} | 2009 | 53.7 | 35.2 | 30.6 | 77.8 | 40.3 | 55.6 | 4 |
| Manchikanti, {An introduction to an evidence-based approach} | 2009 | 88.9 | 44.4 | 52.1 | 52.8 | 54.2 | 87.5 | 5 |
| Easa | 2011 | 81.5 | 35.2 | 54.9 | 46.3 | 13.9 | 44.4 | 4 |
| Manchikanti | 2013 | 77.8 | 66.7 | 64.6 | 77.8 | 29.2 | 83.3 | 5 |
| **Neck Pain w/Headache** | | | | | | | | |
| Sandrini | 2011 | 50.0 | 41.7 | 43.8 | 75.0 | 10.4 | 16.7 | 4 |
| Beithon | 2013 | 88.9 | 75.0 | 64.6 | 75.0 | 68.8 | 95.8 | 6 |
| Douglas | 2014 | 47.2 | 52.8 | 50.0 | 61.1 | 20.8 | 4.2 | 4 |
| Bryans | 2014 | 61.1 | 61.1 | 65.5 | 66.7 | 31.3 | 66.7 | 5 |
| **CAD** | | | | | | | | |
| Magarey | 2004 | 66.7 | 38.9 | 20.8 | 44.4 | 29.2 | 4.2 | 2 |
| Rivett | 2006 | 69.4 | 50.0 | 35.4 | 61.1 | 27.1 | 8.3 | 3 |
| Anderson-Peacock | 2007 | 61.1 | 50.0 | 30.2 | 36.1 | 22.9 | 4.2 | 2 |
| Harrigan | 2013 | 69.4 | 80.6 | 76.0 | 86.1 | 33.3 | 62.5 | 6 |
| Rushton | 2014 | 58.3 | 47.2 | 40.6 | 41.7 | 18.8 | 16.7 | 3 |
