# Supplementary material for: Comparison of CPG’s for the diagnosis, prognosis and management of non-specific neck pain: a systematic review
Source: BMC Musculoskelet Disord. 2019 Feb 14;20:81. doi: 10.1186/s12891-019-2441-3 (PMC6376764; doi:10.1186/s12891-019-2441-3)
Supplement: Supplementary file 5 — Appendix E Combined table for diagnostic recommendations (Outcome measures, Neck pain classification and clinical tests) for general neck pain and whiplash guidelines. (DOCX 17 kb) [file 12891_2019_2441_MOESM5_ESM.docx]

Additional file 5: **APPENDIX E** *DIAGNOSTIC RECOMMENDATIONS AMONG GENERAL NECK PAIN AND WHIPLASH GUIDELINES*

| **Author** | **Year** | **Outcome Measure** | **Duration** | **Neck pain classification** | **Clinical Tests Used** |
| --- | --- | --- | --- | --- | --- |
| **General Neck Pain** | | | | | |
| AAMPGG | 2003 | VAS | None | None | Pain, posture, palpation, joint mobility, proprioception, function |
| New York WC Board | 2008 | VAS | None | Soft tissue: Graded 1-4   1. Minor 2. MSK signs 3. Neuro Signs 4. Fx./Dislocation | ROM, Palpation, DTR’s, Motor and sensory exam, |
| Bone and Joint | 2008 | VAS, NDI | None | 1-4  1: No ADL’s  2: ADL limited  3: Radic  4: Tumor/Fx. | ROM, Palpation, Neck rotation/ext test |
| PAC | 2009 | FCE  Work, Vocational testing, Work tolerance screening | None | None | Hx, PE, Xrays, Lab testing |
| AAMPGG | 2010 | None | Acute:  <3 months  Chronic:  >3 months | None | ROM, All other clinical tests are poor |
| Bono | 2010 | None | None | Radiculopathy classification | DTR, Muscle weakness, Spurlings |
| Brosseau, | 2012 | None | Acute:  <30 days  Subacute:  30-90 days  Chronic:  >90 days | No grade |  |
| Monticone, | 2013 | NDI | Acute:  <1 month  Subacute: 1-3 months  Chronic:  >3 months | 1)Non Specific NP  2)Specific NP  3)NP systemic  4)NP Visceral | ROM, Spurlings Test |
| SIGN | 2013 | None | Chronic:  >12 weeks | No grd given for neck pain | None |
| Bryans | 2014 | NDI | Acute:  <7 days  Subacute: 1 week-3 months  Chronic: >3months | No grd given | None |
| Bussieres | 2016 | None | Acute: 0-3 months  Chronic: >3months | GR 1-4 Bone and Joint  GR 1-4 Whiplash  Does not Cover Grd4 | None |
| Cote | 2016 | None | Acute: 0-3 months  Subacute: 4-6 months  Does not cover >6 months | GR 1-4 Bone and Joint  GR 1-4 Whiplash  Does not Cover Grd4 | None |
| Blanpied | 2017 | NDI  VAS  SF-36  SF-12 | Acute: 0-6weeks  Subacute: 6-12 weeks  Chronic: Greater than 3 months | Neck pain with Mobility deficits  Neck pain with movment coordination impairements (WAD)  Neck pain with headaches (cervicogenic headache)  Neck pain with radiating pain (radicular) | **Neck Pain with Mobility**-ROM, Cervical Flexion-Rotation test (CFRT), Cervical and Thoracic segmental mobility tests  **Neck Pain with Headache**: ROM, CFRT, upper cervical segmental mobility  **Neck Pain with Radiation**: nuerodynamic testing, Spurlings, neck distraction test, Valsalva,  **Neck pain with movment** (WAD)- cranial cervical flexion and neck flexor muscle endurance tests. |
| Kjaer | 2017 | VAS, NPRS, NDI | Acute: <12 weeks | None | None |
| **Whiplash** | | | | | |
| Bekkering | 2003 | None | 0-6 weeks | 0-4 | None |
| Leigh | 2005 | VAS, NDI, Health Status disability, PSFS  Other: RMQ, ODI, UEFI, DASH, LEFS | AC: 0-12 | 0-4 (QTF) | ROM, DTR, Slump, ULTT, VAI testing, Cervical laxity testing |
| Mercer | 2007 | None | AC: 0-2  SA: 2-12  C:>12 | 0-4(QTF) | None |
| TRACs | 2008 | VAS, NDI, SES, CSQ, PSFS, Core whiplash OM, Kessler Psychological distress scale, Impact event scale | AC: 0-12  C: >12 weeks | 0-4 (QTF) | None |
| Davis | 2009 | VAS, NPS, NDI, IES, SF-36 | AC: 0-12 | 0-4 (QTF) | PNF, ULTT, SLR, Slump, UMN, Clonus, Thoracic outlet, Cervical stability, subluxation |
| Bryans | 2010 | None | AC: <1,  SA 1-12,  C: >12 | 0-4 (QTF) | None |
| Moore | 2010 | None | AC:0-2  SA: 2-12  C: >12 | 0-4 (QTF) | ROM, DTR’s PNF (Passive neck flexion), ULTT, SLR, Slump, Clonus  VA insufficiency, Allen, Adsons test, upper cervical stability testing |
| MAA WAD | 2014 | VAS, NDI, IES | AC: 0-12 | 0-4 (QTF) | None |
