# Supplementary material for: Comparison of CPG’s for the diagnosis, prognosis and management of non-specific neck pain: a systematic review
Source: BMC Musculoskelet Disord. 2019 Feb 14;20:81. doi: 10.1186/s12891-019-2441-3 (PMC6376764; doi:10.1186/s12891-019-2441-3)
Supplement: Supplementary file 6 — Appendix F Combined table for prognostics factors for general neck pain and whiplash guidelines (DOCX 20 kb) [file 12891_2019_2441_MOESM6_ESM.docx]

Additional file 6: **Appendix F** *Prognostic factors for general neck pain and whiplash* *guidelines*

| **Author** | **Year** | **Risk/Prognostic Factors** | | | | | | | | | | | | | | |
| --- | --- | --- | --- | --- | --- | --- | --- | --- | --- | --- | --- | --- | --- | --- | --- | --- |
|  |  | **CS** | **IMG** | **ROM** | **SEX** | **VAS** | **NDI** | **PSY** | **CT** | **HHU** | **AGE** | **NPG** | **PNP** | **NEU** | **Other** |  |
| **General Neck Pain** | | | | | | | | | | | | | | | | |
| Anderson-Peacock | 2005 | x | + | + | x | + | x | + | + | x | x | x | + | + | Risk factors given for treatment rather than prognostic factors affecting care |  |
| Bussieres | 2008 | x | + | + | + | + | + | + | + | x | + | + | + | + | Smk, Diagnostic Imaging guideline risk factors |  |
| Childs | 2008 | x | x | x | x | x | x | + | x | x | + | x | + | + | LBP, cycling |  |
| New York WC Board | 2008 | x | + | x | x | x | x | + | x | x | x | x | x | + | Smk, Infection, tumor, Cauda equina, extraspinal disorders. |  |
| Guzman | 2008 | x | x | x | + | x | x | + | + | + | + | + | + | + | Smk, Headache, work related factors, posture, compensation, |  |
| AAMPGG | 2010 | x | x | x | x | x | x | x | + | x | + | x | x | + | Headache, Chronic pain, Thoracic pain, infection, neoplasm, med use |  |
| Monticone | 2013 | x | + | x | x | + | + | + | + | x | + | x | + | + | Rheumatic disease, neoplasms, infections, visceral origin disease, cancer hx, HIV, drugs |  |
| Colorado Division WC | 2014 | x | x | + | x | x | x | + | + | x | + | + | + | + | BMI, Genetic factors not factors |  |
| Cote | 2016 | x | x | x | + | x | x | + | x | x | + | + | + | + | Kinesophobia |  |
| Blanpied | 2017 | + | - | x | + | + | + | + | - | x | - | x | + | x | Post-traumatic stress, pain related catastrophizing, Prior health, Smk, work related factors, sick leave |  |
| Bier | 2018 | x | x | x | x | x | + | x | x | x | x | x | + | x |  |  |
| **Whiplash** | | | | | | | | | | | | | | | | |
| Bekkering et. al, | 2003 | x | x | + | + | x | x | + | x | x | + | x | x | x |  |  |
| Leigh et. al | 2005 | x | x | x | x | + | + | + | + | + | x | x | + | + | Loss of counciousness, multiple areas, Home/work environment, poor coping, structural damage |  |
| TRACsa | 2008 | + | - | + | x | + | + | + | - | x | - | x | x | x | Educational level |  |
| Davis et. al. | 2009 | + | + | + | x | + | + | + | + | x | x | + | + | + | Biomechanical stress, Poor compliance, Re-injury |  |
| Bryans et. al. | 2010 | x | x | x | + | + | x | + | - | + | + | + | + | x | Time since injury, Legal/Comp factors |  |
| Moore et. al. | 2010 | x | + | x | + | + | x | + | + | x | + | x | + | + | Headaches, Comp, Perceptions of work, job context, BP, dizziness |  |

|  |
| --- |

Indicates recommendation without reference citation

|  |
| --- |

Indicates recommendation with RCT or Cohort Study

|  |
| --- |

Indicates recommendation with reference to a systematic review.

+ Recommended -Not recommended I- Insufficient evidence X-Did not mention

CS: Cold sensitivity

IMG: Imaging (MRI, CT, Ultrasound or XRAY) results

ROM: Range of Motion

SEX: Gender

VAS: Visual Analog Scale or Pain Intensity

NDI: Neck Disability Index/self-reported disability

PSY: Psychological Factors

CT-Collision Type/Trauma

HHU: High Healthcare usage/medication

PEF: Pre-existing factors

IMG: Imaging

NPG: Neck pain grade (WAD1-4, Neck pain 1-3)

SMK: Smoking

PNP: Pre-existing neck pain

ED: Education

NEU: Neurological Signs

SMK: Smoking
