# Supplementary material for: Comparison of CPG’s for the diagnosis, prognosis and management of non-specific neck pain: a systematic review
Source: BMC Musculoskelet Disord. 2019 Feb 14;20:81. doi: 10.1186/s12891-019-2441-3 (PMC6376764; doi:10.1186/s12891-019-2441-3)
Supplement: Supplementary file 7 — Appendix G Combined table for psychological prognostic factors for neck pain and whiplash guidelines (DOCX 19 kb) [file 12891_2019_2441_MOESM7_ESM.docx]

Additional file 7: **Appendix G** *Psychological Prognostic Factors for Neck Pain and Whiplash Guidelines*

| **Author** | **Year** | **Psychological Factors** | | | | | | | | | | | | | | | |
| --- | --- | --- | --- | --- | --- | --- | --- | --- | --- | --- | --- | --- | --- | --- | --- | --- | --- |
|  |  | **COP** | **DEP** | **PTS** | **CAT** | **KIN** | **ANX** | **SOM** | **SE** | **JOB** | **FA** | **FAM** | **ATT** | | **SR** | **Other** |  |
| **General Neck Pain** | | | | | | | | | | | | | | | | | |
| Bussieres | 2008 | + | + | + | x | + | x | + | x | x | x | x | x | + | | Psychological stress |  |
| New York WC Board | 2008 | x | + | + | x | x | + | x | x | x | x | x | x | x | | Psychiatric illness and other psychological issues |  |
| Guzman | 2008 | + | x | x | x | + | + | x | x | x | x | x | x | x | |  |  |
| AAMPGG | 2010 | x | x | x | x | x | x | x | x | x | x | x | x | x | | No details given for psychological factors |  |
| Monticone | 2013 | + | + | x | + | + | + | x | x | x | + | x | x | x | | Dysfunctional thoughts |  |
| Colorado Division WC | 2014 | + | + | x | x | x | x | x | x | x | + | x | x | x | | Prior physical/emotional abuse, drug/opiod abuse; chronic pain |  |
| Cote | 2016 | + | + | + | x | + | + | x | x | x | + | x | + | x | | Poor exp. For recovery |  |
| Blanpied | 2017 | x | x | + | + | x | x | x | x | x | x | x | + | + | |  |  |
| Bier | 2018 | + | x | x | x | + | x | x | x | x | x | x | x | x | |  |  |
| **Whiplash** | | | | | | | | | | | | | | | | | |
| Bekkering et. al, | 2003 | + | x | x | x | x | x | x | x | + | x | x | x | | x |  |  |
| Leigh et. al | 2005 | x | + | x | x | x | x | x | x | x | + | x | x | | x | Prefers passive treatment |  |
| TRACsa | 2008 | + | - | x | + | x | + | + | x | - | + | + | + | | + | Diagnostic and treatment issues, |  |
| Davis et. al. | 2009 | x | x | x | x | x | x | x | x | x | x | x | x | | x | High number of complaints, poor compliance |  |
| Bryans et. al. | 2010 | + | + | x | + | + | + | x | + | x | x | x | x | | x |  |  |
| Moore et. al. | 2010 | + | + | + | + | + | + | + | + | + | x | + | + | | x | Clinical behaviors, neg. tx expectations, poor compliance, therapist issues |  |
| MAA | 2014 | + | + | + | + | + | + | + | x | + | x | + | x | | x |  |  |

+ Recommended -Not recommended I- Insufficient evidence X-Did not mention

|  |
| --- |

Indicates recommendation without reference citation

|  |
| --- |

Indicates recommendation with RCT or Cohort Study reference

|  |
| --- |

Indicates recommendation with direct reference to systematic review.

COP- Coping strategies SOM- Somatization

DEP- Depression SE – Low self-efficacy

PTS – Post-Traumatic Stress JOB- Job dissastifaction, work related or compensation factors

CAT – Catastrophization FA- Fear Avoidance

KIN – Kinesiophobia FAM- Family or Social factors

ANX – Anxiety SOM- Somatization ATT- Attitudes and Beliefs about pain. SR- Self-rated injury/disability
