# Supplementary material for: Comparison of CPG’s for the diagnosis, prognosis and management of non-specific neck pain: a systematic review
Source: BMC Musculoskelet Disord. 2019 Feb 14;20:81. doi: 10.1186/s12891-019-2441-3 (PMC6376764; doi:10.1186/s12891-019-2441-3)
Supplement: Supplementary file 8 — Appendix H Combined table for interventional/treatment recommendations for general neck pain and whiplash guidelines (DOCX 30 kb) [file 12891_2019_2441_MOESM8_ESM.docx]

| Additional file 8: **Appendix H** *Interventional Recommendations for Neck Pain and Whiplash Guidelines*  *Recommendations*  **Author** | **Year** | **Interventional Techniques** | | | | | | | | | | | | | | | | |
| --- | --- | --- | --- | --- | --- | --- | --- | --- | --- | --- | --- | --- | --- | --- | --- | --- | --- | --- |
|  |  | **AE** | **Man** | **Mob** | **ET** | **Ed** | **Trc** | **Mmt** | **Sc** | **Ls** | **Us** | **Med** | **Psy** | | **Mass** | **Acu** | **H/C** | **Other** |
| **General Neck Pain** | | | | | | | | | | | | | | | | | | |
| ANAES | 2003 | + | + | + | + | x | - | x | x | - | + | x | x | + | | x | x | + Rest <3days ,Pro tech, stretching  I-HT |
| Anderson-Peacock | 2005 | + | + | + | + | - | + | + | x | + | + | x | x | + | | x | x | +PIL, IP, PEMT  -Mag, occ release |
| Childs | 2008 | + | + | + | x | - Alone | + | + | - | + | x | x | + | x | | x | x | +Rest, return to early ADLS, full recovery adv. |
| New York WC Board | 2008 | + | + | + | + | + | + | x | - | x | + | + | + | x | | + | + | +BF, RC  -HT, ION, DT |
| Bone and Joint Decade (Guzman) | 2009 | + | + | + | - | + | + | x | - | x | - | I | + | I | | x | - | +PEMT, PIL,  -DT, ERG, TRA |
| Oklahoma WC | 2009 | + | + | + | + | + | + | x | - | x | + | + | + | + | | + | + | +BF, ION, DT, HT  -Prolo, Rest <2days |
| AAMPGG | 2010 | + | I | I | I | -Alone +Stay active | I | + | - | x | x | I | I | x | | I | x | +PEMT  I: Spray/Stretch, Microbreaks, gymnastics |
| Brosseau | 2012 | x | x | x | x | x | x | x | x | x | x | x | x | +ST  -LT | | x | x |  |
| SIGN | 2013 | + | + | + | + | + | - | + | x | x | x | + | x | x | | + | x | +PEMT, CBT |
| Monticone | 2013 | + | + | + | + | - | - | + | - | + | + | + | + | - Alone | | + | x | + PEMT |
| Bryans | 2014 | + | + | + | I | x | I | + | x | I | x | x | x | +ST | | x | x |  |
| Colorado Division WC | 2014 | + | + | + | + | + | + | + | - | + | x | + | + | + | | + | x | +BF, RC, DT  -PROLO, ION, |
| Bussieres | 2016 | + | + | + | - | -  Alone | I | + | - | - | x | + | x | -Gr1/2 NP  +Gr3 | | x | x | +Yoga |
| Cote | 2016 | + | + | + | -  Alone;  +  Comb | - Alone | - | + | - | +Gr1/2  -Gr 3 | x | + | -Alone | +Gr1/2 | | - | x | -DT, Qiqong, Yoga |
| Blanpied | 2017 | + | + | + | x | x | + (C) | +  (C) | x | + (C) | x | x | x | x | | +C | x |  |
| Kjaer | 2017 | + | + | + | x | + | + | + | x | x | x | + | x | - | | + | x | -Acu for Radic |
| Bier | 2018 | + | + | + | + | x | + | + | + | + | + | x | + | + | | + | + | Pillows, CBT, KT-tape, Ergonimic Assesment, Neural tissue mang. |
| **Whiplash** | | | | | | | | | | | | | | | | | | |
| Bekkering | 2003 | + | x | x | x | + | x | x | x | x | x | x | + | | x | x | x |  |
| Leigh | 2005 | + | + | + | + | + | x | x | - | x | x | x | + | | x | x | + | +BF, Post. and Erg ed |
| Mercer | 2007 | + | + | + | I | + | x | + | - | x | I | x | + | | I | I | x | I-BF  +STM |
| TRACsa | 2008 | + | + | + | + (A)  –(C) | + | x | + | - | x | x | - | + | | + | x | + | -Surgery, Pillows, rest, Injections |
| Davis | 2009 | + | + | + | + | + | + | + | x | + | + | + | x | | + | + | + | +PEMT, Nutritional sup., Surgery, Inj |
| Moore | 2010 | + | + | + | + TENS  -IFC  -(C) | + | x | + | - | - | - | x | + | | I-(A),(C)  +Sub | I | x | I-PEMT, BF |
| Bryans | 2010 | + | + | + | + | + | x | + | x | x | x | + | + | | x | x | x |  |
| MAA WAD | 2014 | + | + | + | I | + | I | x | x | I | I | + | x | | I | - | I | +KT Tape  -Surgery other than persistent radic  I-Pil, Homeopathy, DT, Mag. Necklaces, Pillows  +TP needling |
| Blanpied | 2017 | + | x | + | +Tens | + | x | + | x | x | x | x | + | | x | x |  |  |
| **Neck Pain w/Headache** | | | | | | | | | | | | | | | | | | |
| Beithon | 2013 | + | - | x | - | + | x | x | x | x | x | + | + | | x | + | x | +BF, herbal therapy, relaxation |
| Bryans | 2014 | +CG | CG  -TTH | +  ALL | x | x | -  TTH | -TTH | x | x | x | x | x | | x | x | x |  |
| Blanpied | 2017 | + | + | + | x | x | x | x | x | x | x | x | x | | x | x | x |  |
| **Radiculopathy** | | | | | | | | | | | | | | | | | | |
| Bono | 2011 | + | - | + | + | x | + | + | x | x | x | - | + | | x | + | x | +surgical |
| Kjaer | 2017 | + | + | + | x | + | + | + | x | x | x | + | x | - | | + | x | -Acu for Radic |
| Blanpied | 2017 | + | + | + | x | + | + (C) | x | +  (A) | x | x | x | x | | x | x | x |  |

+ Recommended -Not recommended I- Insufficient evidence X-Did not mention

|  |
| --- |

Indicates recommendation without reference citation

|  |
| --- |

Indicates recommendation with RCT or Cohort Study

|  |
| --- |

Indicates recommendation with reference to a systematic review.

AE: Active Exercise SC: Soft Collar

MAN: Manipulation Ls: Laser

MOB: Mobilization Us: Ultrasound

ET: Electrotherapy Med: Medication

ED: Education Psy: Psychological Interventions

Trc: Traction Mass: massage

Mmt: Multi-Modal Therapy ACU: Acupuncture/Dry Needling

RC: Rigid collar; PEMT: Pulsed Magnetic Therapy,

PIL: Pillow; ERG: Ergonomic interventions;

TRA: Fitness Training BF: Biofeedback

Mig: Migraine CBT: Cognitive Behavioral Therapy

TTH: Tension-Type Headache HT: Hydrotherapy/Aquatic therapy

PEMT: Pulsed Magnetic Therapy H/C: Heat/Cold

DT: Diathermy ION: Iontophoresis

CG: Cervicogenic (C): Chronic (A) Acute
