# Supplementary material for: Comparison of CPG’s for the diagnosis, prognosis and management of non-specific neck pain: a systematic review
Source: BMC Musculoskelet Disord. 2019 Feb 14;20:81. doi: 10.1186/s12891-019-2441-3 (PMC6376764; doi:10.1186/s12891-019-2441-3)
Supplement: Supplementary file 9 — Appendix I Imaging. Combined table for imaging recommendations for all guidelines (DOCX 17 kb) [file 12891_2019_2441_MOESM9_ESM.docx]

Additional file 9: **APPENDIX I: Imaging**

| ***Author*** | ***Year*** | ***CCSR*** | ***MRI*** | ***CT*** | ***XRAY*** | ***EMG*** | ***Other*** |
| --- | --- | --- | --- | --- | --- | --- | --- |
| ***General Neck Pain*** | | | | | | | |
| *French* | *2003* | *+* | *+* | *+* | *+* |  |  |
| *Bussieres* | *2008* | *+* | *+ (ligament.*  */neurological signs)* | *+ (CCSR)*  *+Vertebral Artery Dissection* | *-(CCSR) + (CCSR)*  *+Neurological signs*  *+Worse pain >4weeks*  *+ Red flag symptoms.* |  |  |
| *Childs* | *2008* | *+* | *-*  *+Chronic WAD* | *-* | *-* |  | *+Chronic WAD* |
| *New York WC* | *2008* |  | *+Cord Injury* | *+Cord Injury* | *+* | *+* | *+Myelography; CT mylography, lineal tomography, bone scan, fluoroscopy*  *-Dx inj, discography, thermography,* |
| *Bone and Joint* | *2008* | *+* | *+Grd 3,4* | *+Grd 3,4* | *-* | *I* | *+bone scan, inf markers grd 4*  *-blood tests, inj discography,* |
| *PAC* | *2009* |  | *++* | *+* | *+* | *-* | *+Myelography, Bone Scan, Gallium Scan*  *+Dx Inj*  *-Thermography, EvP,* |
| *AAMPG* | *2010* | *+* | *+(Signs of serious pathology)* | *+if X-ray (+)* | *-acute*  *+Trauma* | *X* |  |
| *Bono* | *2010* | *X* | *++* | *+Myelography if MRI +* | *X* | *I* | *+Nerve block (SNRB)* |
| *Newman* | *2014* | *X* | *-normal X-ray*  *+Neuro signs, DJD changes, old trauma w/neuro signs, disc margin destruction or bone lesion infection or CA* | *-*  *+prior C-spine surgery* | *+Chronic NP, history of cancer, neck surgery* | *X* |  |
| *Monticone* | *2013* | *X* | *+Radicular* | *+Radicular* | *+Radicular* | *X* | *-surface electromyography*  *+Needle for radiculopathy*  *-BT* |
| *Cote* | *2016* | *+* | *X* | *X* | *X* | *X* | *X* |
| *Blanpeid* | *2017* | *+* | *+Radicular, mylopathy* | *+(Radicular if no MRI)* | *-Routine* | *X* | *X* |
| ***Whiplash*** | | | | | | | |
| *TRACs* | *2008* | *+* | *x* | *x* | *x* | *x* |  |
| *Davis* | *2009* | *X* | *+* | *+* | *+* | *+* | *Kinetic MRI, SPECT scan, videoflouroscopy* |
| *Moore* | *2010* | *X* | *+WAD4* | *+WAD4* | *+WAD4* |  |  |
| *MAA* | *2014* | *+* | *+WAD3* | *+WAD3* | *+WAD3* | *+WAD3* | *+EEG-WAD3* |
| ***Neck Pain w/Headache*** | | | | | | | |
| *Sandirini* | *2011* | *X* | *+Trigeminal autonomic cephalalgia, atypical headache* | *-* | *-* | *-* | *+EEG if epilepsy*  *-Hemipilgic or basilar mig,*  *-PET or SPECT scan* |
| *Douglas* | *2014* |  | *+Chronic*  *++Chronic w/new neuro deficit*  *, exertion or sex* | *++Sudden onset +Post traumatic* | *x* | *x* | *++CTA/MRA Suspect VAD*  *+Arteriography* |
| **CAD** |  |  |  |  |  |  |  |
| *Harrigan* | *2013* | *X* | *+MRA* | *+CTA* | *X* | *X* | *++Cathetor Angiography (Gold Standard)* |

+ Recommended -Not recommended I- Insufficient evidence X-Did not mention

CCSR- Canadian Cervical Spine Rule

EEG- Electroencephalogram
