# Supplementary material for: Comparison of CPG’s for the diagnosis, prognosis and management of non-specific neck pain: a systematic review
Source: BMC Musculoskelet Disord. 2019 Feb 14;20:81. doi: 10.1186/s12891-019-2441-3 (PMC6376764; doi:10.1186/s12891-019-2441-3)
Supplement: Supplementary file 10 — Appendix J Combined table for diagnostic recommendations among interventional focused guidelines (DOCX 14 kb) [file 12891_2019_2441_MOESM10_ESM.docx]

Additional file 10: **APPENDIX J** *DIAGNOSTIC RECOMMENDATIONS AMONG INTERVENTIONAL-FOCUSED GUIDELINES*

| **Author** | **Year** | **Diagnostic Techniques** | | | | |
| --- | --- | --- | --- | --- | --- | --- |
|  |  | **Disco** | **SNRB** | **TESI** | **FJNB** | **Other** |
| Boswell | 2005 | I | + | + | + |  |
| Boswell | 2005 | I | + | + | + |  |
| Manchikanti, {Evidence-Based Guidelines} | 2009 | I | X | X | + |  |
| Manchikanti, {Review of Therapeutic Interventions} | 2009 | I | + | X | + |  |
| Manchikanti,  {Review of therapeutic interventions} | 2009 | I | X | X | + |  |
| Manchikanti,  {An algorithmic approach} | 2009 | I | + | + | + | Imaging (+) (Situationally) |
| Manchikanti | 2013 | I | X | X | + |  |

+ Recommended -Not recommended I- Insufficient evidence X-Did not mention

Disco=Discography

SNRB=Selective Nerve Root Blocks

TESI= Transforaminal Epidural Steroid Injections

FJNB= Facet Joint Nerve Block
