# Supplementary material for: Comparison of CPG’s for the diagnosis, prognosis and management of non-specific neck pain: a systematic review
Source: BMC Musculoskelet Disord. 2019 Feb 14;20:81. doi: 10.1186/s12891-019-2441-3 (PMC6376764; doi:10.1186/s12891-019-2441-3)
Supplement: Supplementary file 11 — Appendix K Combined table for all invasive techniques recommended by interventional and non-interventional focused guidelines (DOCX 20 kb) [file 12891_2019_2441_MOESM11_ESM.docx]

Additional file 11: **APPENDIX K** *Invasive Techniques Recommended by Interventional Focused Guidelines*

| ***Author*** | | | | ***Year*** | | ***Invasive Techniques*** | | | | | | | | | | | | | | | | | | | | | |
| --- | --- | --- | --- | --- | --- | --- | --- | --- | --- | --- | --- | --- | --- | --- | --- | --- | --- | --- | --- | --- | --- | --- | --- | --- | --- | --- | --- |
|  |  |  |  |  |  | ***ESI*** | | ***PA*** | | ***IIIS*** | | ***FJI*** | | | ***MBB*** | | ***MBN*** | | | ***RN*** | | ***VB*** | | ***Other*** | | | |
| ***Invasive techniques from Interventional-Focused Guidelines*** | | | | | | | | | | | | | | | | | | | | | | | | | | | |
| *Boswell* | | | | *2005* | | *+* | | *+* | | *+* | | *-* | | | *+* | | *+* | | | *+* | | *x* | |  | | | |
| *Boswell* | | | | *2007* | | *+* | | *+* | | *+* | | *-* | | | *+* | | *+* | | | *+* | | *+* | |  | | | |
| *Manchikanti* | | | | *2008* | | *+* | | *+* | | *+* | | *x* | | | *+* | | *x* | | | *+* | | *x* | |  | | | |
| *Manchikanti, {Evidence-Based Guidelines}* | | | | *2009* | | *+* | | *+* | | *+* | | *x* | | | *+* | | *x* | | | *+* | | *x* | |  | | | |
| *Manchikanti, {Review of Therapeutic Interventions}* | | | | *2009* | | *+* | | *+* | | *+* | | *I* | | | *+* | | *x* | | | *+* | | *x* | |  | | | |
| *Manchikanti, An {Algorithmic Approach}* | | | | *2009* | | *+* | | *+* | | *+* | | *I* | | | *+* | | *x* | | | *+* | | *x* | |  | | | |
| *Easa* | | | | *2011* | | *+* | | *x* | | *x* | | *x* | | | *+* | | *x* | | | *x* | | *x* | |  | | | |
| *Manchikanti* | | | | *2013* | | *+* | | *+* | | *I* | | *I* | | | *+* | | *x* | | | *+* | | *x* | |  | | | |
| ***Invasive techniques from non-interventional focused guidelines*** | | | | | | | | | | | | | | | | | | | | | | | | | | | |
| ***Author*** | ***Year*** | ***Invasive Techniques*** | | | | | | | | | | | | | | | | | | | | | | | | | |
|  |  | ***FJI*** | ***PA*** | | ***SI*** | | ***MBN*** | | ***Prolo*** | | ***BTX*** | | ***RFA*** | ***BI*** | | ***PRDD*** | | ***DR*** | ***Surg*** | | ***NB*** | | ***Etan*** | | ***PD*** | ***TPI*** | ***Other*** |
| *New York WC Board* | *2008* | *I* | *-* | | *I* | | *I* | | *-* | | *x* | | *x* | *x* | | *-* | | *+* | *+*  *Cert* | | *+* | | *x* | | *+*  *Cert* | *+* |  |
| *Bone and Joint Decade (Guzman)* | *2009* | *x* | *x* | | *+* | | *x* | | *x* | | *x* | | *x* | *x* | | *x* | | *x* | *I* | | *x* | | *x* | | *x* | *x* |  |
| *Oklahoma WC* | *2009* | *+* | *x* | | *+* | | *+* | | *-* | | *x* | | *x* | *x* | | *-* | | *x* | *+ Cert* | | *+* | | *x* | | *-* | *+* |  |
| *Colorado Division WC* | *2014* | *-* | *-* | | *-* | | *+* | | *-* | | *I* | | *-* | *-* | | *-* | | *+* | *+ Cert* | | *x* | | *-* | | *+ Cert* | *I* | *- rhBMP-2* |
| *Bono* | *2011* | *x* | *x* | | *I* | | *x* | | *x* | | *x* | | *x* | *x* | | *x* | | *-* | *+* | | *x* | | *x* | | *x* | *x* | *+ Cerv. Decom.*  *+ ACD and Fusion* |
| *TRACsa* | *2008* | *x* | *x* | | *-* | | *x* | | *x* | | *x* | | *x* | *x* | | *x* | | *x* | *-* | | *x* | | *x* | | *x* | *x* | *+ Medrol* |
| *MAA WAD* | *2014* | *x* | *x* | | *-* | | *x* | | *x* | | *-* | | *x* | *x* | | *x* | | *x* | *+*  *Cert* | | *x* | | *x* | | *x* | *-* |  |
| *Bussieres* | *2016* | *x* | *x* | | *x* | | *x* | | *x* | | *-* | | *x* | *x* | | *x* | | *x* | *x* | | *x* | | *x* | | *x* | *x* |  |
| *Cote* | *2016* | *x* | *x* | | *x* | | *x* | | *x* | | *-* | | *x* | *x* | | *x* | | *x* | *x* | | *x* | | *x* | | *x* | *x* |  |

|  |
| --- |

Indicates recommendation without reference citation

|  |
| --- |

Indicates recommendation with RCT or Cohort Study

|  |
| --- |

Indicates recommendation with direct reference to systematic review.

+ Recommended -Not recommended I- Insufficient evidence X-Did not mention

PA=Percutaneous Adhesiolysis PD=Percutaneous Discectomy

IIIS=Implantable intrathecal infusion systems Etan=Etanercept

IFJI= Intraarticular Facet joint injections TPI=Trigger Point Injections

MBB=Medial Branch Blocks ACD= Anterior Cervical Discectomy

MBN=Medial Branch Neurotomy Cert=Certain Procedures/On Certain Occasions

Radiofrequency Neurotomy =RN NB = Nerve Blocks

VB=Vertebroplasty

SI=Steroid Injections

Prolo=Prolotherapy

BTX=Botox

RFA=Radiofrequency Ablation

BI=Biacuplasty

PRDD = Percutaneous radiofrequency disc decompression

DR=Disc Replacement
