# Supplementary material for: Comparison of CPG’s for the diagnosis, prognosis and management of non-specific neck pain: a systematic review
Source: BMC Musculoskelet Disord. 2019 Feb 14;20:81. doi: 10.1186/s12891-019-2441-3 (PMC6376764; doi:10.1186/s12891-019-2441-3)
Supplement: Supplementary file 12 — Appendix L Combined table for VAI risk factors and testing procedures (DOCX 14 kb) [file 12891_2019_2441_MOESM12_ESM.docx]

Additional file 12: **Appendix L**: Cervical Arterial Dysfunction (CAD) Risk Factors and Testing Procedures

| Guideline | Risk Factors | Testing |
| --- | --- | --- |
| Magarey, 2004 | Provocative testing (sustained end range rotation), Trauma, Neurological changes, Nausea | Sustained end range rotation only |
| Rivett, 2006 | Previous VBI, Visual disturbances, dysarthria, dysphagia, diplopia, drop attacks, nausea, lightheadedness and fainting, disorientation or anxiety, hearing disturbances/tinnitus, facial paraesthia or anaesthesia, pallor, tremors, sweating, other neurological symptoms | Quadrant testing/End range rotation  Pre-Manipulative position |
| Anderson-Peacock, 2007 | Hx. of CAD, Active CAD, Spinal cord injury, carotid stenosis, cardiac disease/abnormalities, integumentary lesions, inflammatory arthritides, mediolytic arteriopathy, pain with patient position, known malingering, somatoform w/no physical involvement, hypochondriasis, neurolgic difficulties, pathology resulting in bone/joint/ligament weakening/malformation, throid tumor, malignancy C-spine, connective tissue disorders, chronic calcium deposit, gout, failed back surgery | None |
| Harrigan, 2013 | Treatment: Anti-coagulant/Antiplatlet therapy, | Cathetor Angiography (Gold Standard), CTA, MRA |
| Rushton, 2014 | Risk factors: Hypertension, instability of cranial region  Mid-upper cervical pain; occipital headache; Acute onset of pain described as “unlike  any other”. Hindbrain transient ischaemic attack (dizziness, diplopia, dysarthria, dysphagia, drop attacks, nausea, nystagmus, facial numbness, ataxia, vomiting, hoarseness, loss of short term memory, vagueness, hypotonia/limb weakness [arm or leg], anhidrosis [lack of facial sweating], hearing disturbances, malaise, perioral dysthaesia, photophobia, papillary changes, clumsiness agitation); Cranial nerve dysfunction;  Hindbrain stroke (e.g. Wallenberg’s syndrome, locked-in syndrome). Hypertension, Inc. BP, cervical Instability | Cervical instability tests (lacking evidence)  Pre-Manipulative testing/end range rotation  Palpation |
